# Projections of diabetes mellitus in Japan through 2040: a bayesian age-period-cohort analysis of the national health and nutrition survey population

**DOI:** 10.1016/j.lanwpc.2026.101988

**Published:** 2026-09-22

**Authors:** Rajib Mondal, Soshiro Ogata, Eri Kiyoshige, Yuriko Nakaoku, Kiyomasa Nakatsuka, Tomomi Watanabe, Kanako Teramoto, Aya Kadota, Katsuyuki Miura, Chris Kypridemos, Yusuke Yoshikawa, Kunihiro Nishimura

**Affiliations:** a Department of Preventive Medicine and Epidemiology, National Cerebral and Cardiovascular Center, Osaka, Japan; b Department of Preventive Medicine, Shiga University of Medical Science, Shiga, Japan; c Department of Biostatistics, National Cerebral and Cardiovascular Center, Osaka, Japan; d Department of Public Health, Policy and Systems, University of Liverpool, Liverpool, United Kingdom; e Department of Data Science, Hyogo Medical University, Hyogo, Japan

**Keywords:** Bayesian age-period-cohort, Bayesian age-period-cohort modeling and prediction, Projection modeling, Diabetes trends

## Abstract

**Background:**

Accurate projection of the burden of diabetes mellitus (DM) requires consideration of age, period, and cohort (APC) effects underlying prevalence trends. We estimated projected trends in DM prevalence and case numbers in Japan from 2020 until 2040, by sex, using the Bayesian APC (BAPC) model.

**Methods:**

We employed Bayesian age-period-cohort modeling and prediction (BAMP), using observed populations (aged 40 years or older) and DM cases from the National Health and Nutrition Survey from 2003 to 2019, to project national-level DM prevalence and cases from 2020 to 2040 using the national-level projected population until 2040. We further compared BAPC's predictive performance with that of conventional prediction model 1 with a constant prevalence rate and conventional prediction model 2 with a monotonically changing prevalence rate, using root mean square error (RMSE) between observed and projected case numbers for model validation during 2015–2019.

**Findings:**

The prevalence (95% credible intervals) of DM in Japan was projected to increase from 16.4% (12.3%–21.6%) in 2020 to 18.1% (6.4%–40.8%) in 2040 in men by BAPC, while it was projected to decline from 9.6% (6.8%–13.3%) to 7.7% (2.3%–22.7%) in women, variations primarily suggested by age effects and cohort effects. DM cases (95% credible intervals) are expected to increase from 6,070,000 (4,530,000–7,980,000) in 2020 to 6,336,000 (2,230,000–14,290,000) in 2040 in men, while they were expected to decline from 3,995,000 (2,840,000–5,540,000) to 3,032,000 (910,000–8,910,000) in women. The BAPC demonstrated the best predictive performance with lower RMSE (men, 40.1; women, 11.5), compared to conventional model 1 (men, 51.3; women, 20.8) and conventional model 2 (men, 42.4; women, 23.1).

**Interpretation:**

This study suggests that Japan is expected to experience increases in the prevalence and number of DM cases from 2020 to 2040, particularly among men, given the APC factors.

**Funding:**

This research was supported by JSPS KAKENHI Grant.

**Research in context**

**Evidence before this study**

• The Global Burden of Disease study projected the prevalence of diabetes mellitus (DM) to increase from 5.9% in 2021 to 6.7% in 2050, employing a Bayesian meta-regression forecasting model using body mass index and calendar year (temporal factor) as the predictors.
• However, projections of DM prevalence trends accounting for age-period-cohort (APC) effects are scarce globally, including in the Western Pacific region.

**Added value of this study**

• The current Bayesian age-period-cohort (BAPC) prediction model accounted for the age effects (across different age groups), period effects (over the calendar time), and cohort effects (birth cohort in a particular period) components and explored a precise projection of the number of DM cases and prevalence at the national level in Japan using nationally representative samples.
• The BAPC indicated that DM prevalence (95% credible intervals) at the national level in Japan was projected to increase from 16.4% (12.3%–21.6%) in 2020 to 18.1% (6.4%–40.8%) in 2040 in men, while it was projected to decline from 9.6% (6.8%–13.3%) to 7.7% (2.3%–22.7%) in women, variations primarily driven by age effects and cohort effects.
• DM cases (95% credible intervals) are expected to increase from 6,070,000 (4,530,000–7,980,000) in 2020 to 6,336,000 (2,230,000–14,290,000) in 2040 in men, while they were expected to decline from 3,995,000 (2,840,000–5,540,000) to 3,032,000 (910,000–8,910,000) in women.

**Implications of all the available evidence**

• Our study urges reform and implementation of effective public health policies to challenge the potential future burden of DM in Japan. It may include strengthening the healthcare system for screening and early detection of DM in primary care, meeting health needs (especially for older adults and men), monitoring the burden of DM, and taking measures to prevent DM and its complications (e.g., lifestyle interventions).

## Introduction

Diabetes mellitus (DM) is one of the major causes of death and disability globally.^1^ The public health, societal, and economic burden of DM is widespread and rapidly increasing.^1^, ^2^, ^3^ In Japan, DM has become a major public health concern, with a prevalence of around 8–9%.^4^, ^5^, ^6^ The Global Burden of Disease (GBD) study estimated the DM cases to increase from 15 million (age-standardized prevalence of 5.9%) in 2021 to over 16 million (age-standardized prevalence of 6.7%) in 2050.^2^ However, accurate projection of DM burden requires consideration of age, period, and cohort (APC) effects underlying prevalence trends.

The GBD study used hierarchical Bayesian meta-regression to forecast DM case numbers and prevalence, incorporating body mass index and calendar year as predictors.^2^ The Health Japan-21 project used a trend-analysis tool to forecast sex- and age-specific prevalence.^7^ However, relying on conventional or limited-span predictive models and heterogeneous DM-defining methods, including the use of casual serum glucose, may limit the precise projection of DM prevalence and the distinction of underlying APC effects in the prevalence trend. Cohort effects have been demonstrated as a key component in the Bayesian projection analysis of trends of cardiovascular disease risk factors^8^^,^^9^ and cardiovascular mortality^10^ in Japan. These limitations warrant further refinement in understanding trends in DM prevalence by using more advanced, well-parameterized projection models and robust criteria for defining DM. Such investigations are crucial for gaining better insight into the potential future burden and for addressing the situation with long-term policy implications. Bayesian age-period-cohort (BAPC) modeling accounts for age effects (across age groups), period effects (over calendar time), and cohort effects (birth cohort in a particular period) to gain insight into their impacts on the projections.^11^, ^12^, ^13^

A prior study projected trends in serum glucose levels using BAPC from 1989 to 2018 among Japanese who participated in the National Health and Nutrition Survey (NHNS).^8^ However, studies exploring trends and projecting DM prevalence while accounting for APC effects are scarce globally and in the Western Pacific region. We investigated projected trends in the prevalence of DM at the national level in Japan between 2020 and 2040, both overall and by sex, accounting for APC effects utilizing state-of-the-art BAPC modeling and evidence from nationally representative NHNS data during 2003–2019.

## Methods

### Study design

We used NHNS data by year, age, and sex between 2003 and 2019 to develop prediction models for the projected prevalence of DM from 2020 to 2040. We employed three statistical methods to demonstrate that the APC effects and algorithm selection are essential for obtaining a reliable projected prevalence of DM. Firstly, we employed the BAPC model, using Bayesian age-period-cohort modeling and prediction (BAMP), which incorporates APC effects. Secondly, we employed two conventional models: model 1, with a constant prevalence, and model 2, with monotonic changes, both derived from Poisson regression models.

### Data source

Initiating after World War II as the National Nutrition Survey, it was restructured and renamed NHNS in 2003. The NHNS provides a database for noncommunicable disease risk factors in Japan. Using a stratified and two-stage cluster sampling method, this cross-sectional household examination survey annually collects comprehensive data on diet, physical activity, lifestyle, anthropometric measures, clinical profiles, and laboratory measurements from nationwide representative samples of approximately 8,000–11,000 individuals aged 1 year and older.^8^^,^^14^ We used participants’ glycemic data for this study.

### Outcome measures

Our primary outcome was the projected national-level prevalence of DM from 2020 to 2040, further stratified by sex. Secondarily, we projected the number of DM cases using similar approaches. We defined DM by the National Glycohemoglobin Standardization Program (NGSP) glycated hemoglobin A1c (HbA1c) ≥6.5%, or self-reported use of glucose-lowering medication.^15^ Notably, our DM definition corresponded to the official NHNS's ‘strongly suspected diabetes’ category,^16^, ^17^, ^18^ rather than strictly diagnosed diabetes. This definition is particularly suitable for capturing population-level burden of diabetes, thereby appropriate for its population-level surveillance and long-term projections. HbA1c was measured by latex agglutination immunoassays until 2011, as per the Japanese Diabetes Society (JDS) guidelines. The JDS HbA1c values were then converted to the NGSP values using the following formula: HbA1c% (NGSP) = (1.02 × HbA1c% (JDS) + 0.25).

This DM definition allowed us to validate our results externally, additionally by directly comparing them with the official NHNS-reported prevalence of the ‘strongly suspected diabetes’ category in 2022–2024.^16^, ^17^, ^18^ This process also highlights the predictive ability of our models for those years. Notably, the NHNS was not conducted in 2020 and 2021 due to the coronavirus disease-19 pandemic, and the complete data for 2022–2024 are not yet available.

### Statistical analysis

Data on DM prevalence and case numbers were summarized using descriptive statistics, including frequency and percentage. All data analyses were conducted by the statistical software R version 4.3.1.^19^

### BAPC statistical modeling

We developed BAPC modeling employing the R *bamp* package,^20^ fitting the population and DM case data stratified by sex and 9 age groups (40–44, 45–49, 50–54, 55–59, 60–64, 65–69, 70–74, 75–79, and ≥80 years). In practice, a similar approach was previously applied to projections of cardiovascular disease mortality in the United Kingdom,^21^ the United States,^22^ and Japan.^10^ BAMP assumes a hierarchical model with a binomial model for event counts, given the population at risk, at the first stage, followed by assigning smoothing priors for APC parameters using first- and second-order random walks (RW1 or RW2).^11^^,^^20^^,^^23^

The BAPC model assumes the logit risk of incidence or event (i.e., mortality) in a specific age group and period follows a linear combination of an intercept and the APC parameter effects. In addition, APC effects can be a constant or linear time trend (i.e., RW1 or RW2) with or without heterogeneity,^11^^,^^20^^,^^23^ which allowed us to consider various model structures. Among them, the best model structure for total and by sex was determined by root mean square error (RMSE). We used age-specific national-level population sizes from 2020 to 2040 (projected) in the BAPC model to derive the projected national-level DM prevalence and corresponding case numbers. We reported the age-specific and age-aggregated projected crude prevalence and case numbers in median with 95% credible intervals (CI) (i.e., 2.5th and 97.5th percentiles), derived from the Markov chain Monte Carlo (MCMC) posterior distributions of the BAPC models. Since age, period, and cohort are linearly dependent (cohort = period–age) and their linear components (slopes) are inherently non-identifiable in the APC model, interpretation relied on identifiable non-linear components (curvatures) and projected trends dependent on prior distributions. Detailed methods are described in Supplementary Methods.

### Conventional prediction models

In the conventional prediction model 1 with a constant DM prevalence, we projected age-specific future prevalence, assuming a constant prevalence equal to that observed in the last year of the observed data (2019 in this study) for each age group. In the conventional prediction model 2 with monotonic changes in DM prevalence, we projected age-specific future prevalence using prevalences that monotonically changed by the mean percentage change in each age group, estimated from Poisson regression models of the observed data from 2003 to 2019.

### Comparison of BAPC and conventional models

We compared the predictive abilities of three models by RMSE. First, we split the dataset into a training dataset (2003–2014) and a validation dataset (2015–2019). Second, we fitted the models to the training dataset to predict overall age-aggregated DM case numbers (projected values) throughout the validation period. Third, we calculated overall age-aggregated observed case numbers (observed values) using similar approaches. Fourth, differences between projected and observed values were summarized using RMSE, calculated as the square root of (observed values—projected values)^2^. A lower RMSE value highlights better predictive accuracy. However, we predefined BAPC as our primary model, owing to its APC features, comprehensiveness, and robustness in projection modeling. We further reported RMSE for predictive performance of BAPC and conventional models, comparing the predicted prevalence of DM with the official NHNS-reported prevalence across 2022–2024 to strengthen our external validity.

The derivation of other national-level DM prevalence is described in the Supplementary Methods.

Additionally, we estimated age-standardized DM prevalence by the direct method using the 2015 Japan Standard Population. We weighed the national-level age-specific prevalence by the corresponding Standard Population weights. The weighted values were then summed and divided by the total Standard Population to obtain age-standardized prevalence.

### Decomposition analysis

We further conducted decomposition analysis of BAPC-derived projected changes in DM prevalence into contributions from population aging (changes in the demographic age structure) and age-specific prevalence changes, with their interaction.^24^ Annual DM prevalence was calculated as the population-weighted average of age-specific prevalence estimates, based on national population projections for 9 age groups.

### Prior distributions in BAPC and sensitivity analysis

For random-walk components’ smoothing priors, we specified Gamma priors on precision parameters for each APC component as follows–G(a,b). The higher parameters imply stronger smoothing. The hyperparameters G(a,b) were derived assuming a plausible range of differences between adjacent levels, expressed on a log scale of ±δ (delta). In our main analysis, we adopted priors with ±δ values of log (1.5) for all APC effects.

We conducted our sensitivity analysis using 2 strategies. First, we considered a set of stronger smoothing prior assumptions with higher hyperparameters to ensure the most plausible predictions. Finally, we used a prior ±δ value of log (2.0) for the age effect, while keeping the period and cohort effects unchanged. Because other prior combinations yielded higher RMSE values, reduced converged models, and non-plausible projection estimates. Details are in Supplementary Methods. Second, NHNS's expanded surveys in 2012 and 2016 were conducted with approximately 4 times the population size as in other usual survey cycles, which may unduly drive estimates of period and cohort effects. Also, NHNS survey weights were not considered in this current analysis. Therefore, we repeated the analysis with down-samples of one-quarter of the population and cases, deliberately dividing the age-specific population and case numbers by 4. We used the same prior ±δ value of log (1.5) as in the main analysis and additionally considered a set of Bayesian parameter combinations for down-samples. We further externally validate the projections by calculating RMSE compared with the official NHNS-reported DM prevalence across 2022–2024 (details in Supplementary Methods).

### Ethics approval

We used NHNS anonymized and secondary data, which contained no personal information about the participants. We accessed NHNS data from the Ministry of Health, Labor and Welfare, employing standard procedure. Hence, the Japanese Ethical Guidelines and Ethical Guidelines for Medical and Biological Research Involving Human Subjects do not apply to our study. Therefore, our study is exempt from the eligibility criteria for institutional ethical approval.

### Role of the funding source

The funding source was public research funding and had no role in study design or conduct, data collection, data analysis, manuscript preparation, or review.

## Results

### Observed DM prevalence

Table 1 describes trends in the observed prevalence of DM from 2003 to 2019 by sex and age group. In general, DM prevalence increased over the years at the national level, from 11.2% in 2003 to 12.5% in 2019. We observed consistent rising trends in men (from 13.6% to 16.1%) and women (from 9.6% to 10.0%). This rising prevalence was particularly notable in men aged 65 years and older and women aged 75 years and older. Details with population and DM case data are available in Supplementary Table S1, Supplementary Table S2, Supplementary Table S3, and Fig. 1.

**Table 1:** Trends in age-specific observed crude prevalence (%) of diabetes mellitus in NHNS participants and age-aggregated observed prevalence at the national level, Japan, from 2003 to 2019.

Population	2003			2010			2019		
	n	Case	Prevalence	n	Case	Prevalence	n	Case	Prevalence
Total									
40–44 years	333	13	3.9	242	14	5.8	144	5	3.5
45–49 years	409	22	5.4	241	11	4.6	182	7	3.8
50–54 years	541	47	8.7	293	26	8.9	173	14	8.1
55–59 years	524	49	9.4	356	33	9.3	177	17	9.6
60–64 years	579	80	13.8	494	79	16.0	215	18	8.4
65–69 years	617	99	16.0	532	87	16.4	363	67	18.5
70–74 years	521	96	18.4	436	82	18.8	360	77	21.4
75–79 years	342	65	19.0	313	66	21.1	281	64	22.8
≥80 years	291	33	11.3	288	46	16.0	247	43	17.4
aNational level			11.2			12.7			12.5
Men									
40–44 years	118	6	5.1	95	11	11.6	48	2	4.2
45–49 years	150	11	7.3	80	3	3.8	67	4	6.0
50–54 years	208	27	13.0	122	16	13.1	59	7	11.9
55–59 years	194	24	12.4	135	22	16.3	70	13	18.6
60–64 years	248	40	16.1	231	46	19.9	84	8	9.5
65–69 years	262	53	20.2	230	47	20.4	165	44	26.7
70–74 years	238	48	20.2	192	42	21.9	164	46	28.0
75–79 years	144	27	18.8	139	35	25.2	133	32	24.1
≥80 years	114	18	15.8	129	19	14.7	107	22	20.6
aNational level			13.6			15.9			16.1
Women									
40–44 years	215	7	3.3	147	3	2.0	96	3	3.1
45–49 years	259	11	4.2	161	8	5.0	115	3	2.6
50–54 years	333	20	6.0	171	10	5.8	114	7	6.1
55–59 years	330	25	7.6	221	11	5.0	107	4	3.7
60–64 years	331	40	12.1	263	33	12.5	131	10	7.6
65–69 years	355	46	13.0	302	40	13.2	198	23	11.6
70–74 years	283	48	17.0	244	40	16.4	196	31	15.8
75–79 years	198	38	19.2	174	31	17.8	148	32	21.6
≥80 years	177	15	8.5	159	27	17.0	140	21	15.0
aNational level			9.6			10.6			10.0

NHNS, National Health and Nutrition Survey.

The definition of diabetes corresponded to the official NHNS's ‘strongly suspected diabetes' category (ascertained by HbA1c% ≥6.5, or medication use), rather than strictly diagnosed diabetes.

a Age-specific prevalence data from NHNS participants were applied to national-level population data to estimate the age-aggregated prevalence at the national level.

**Fig. 1:**
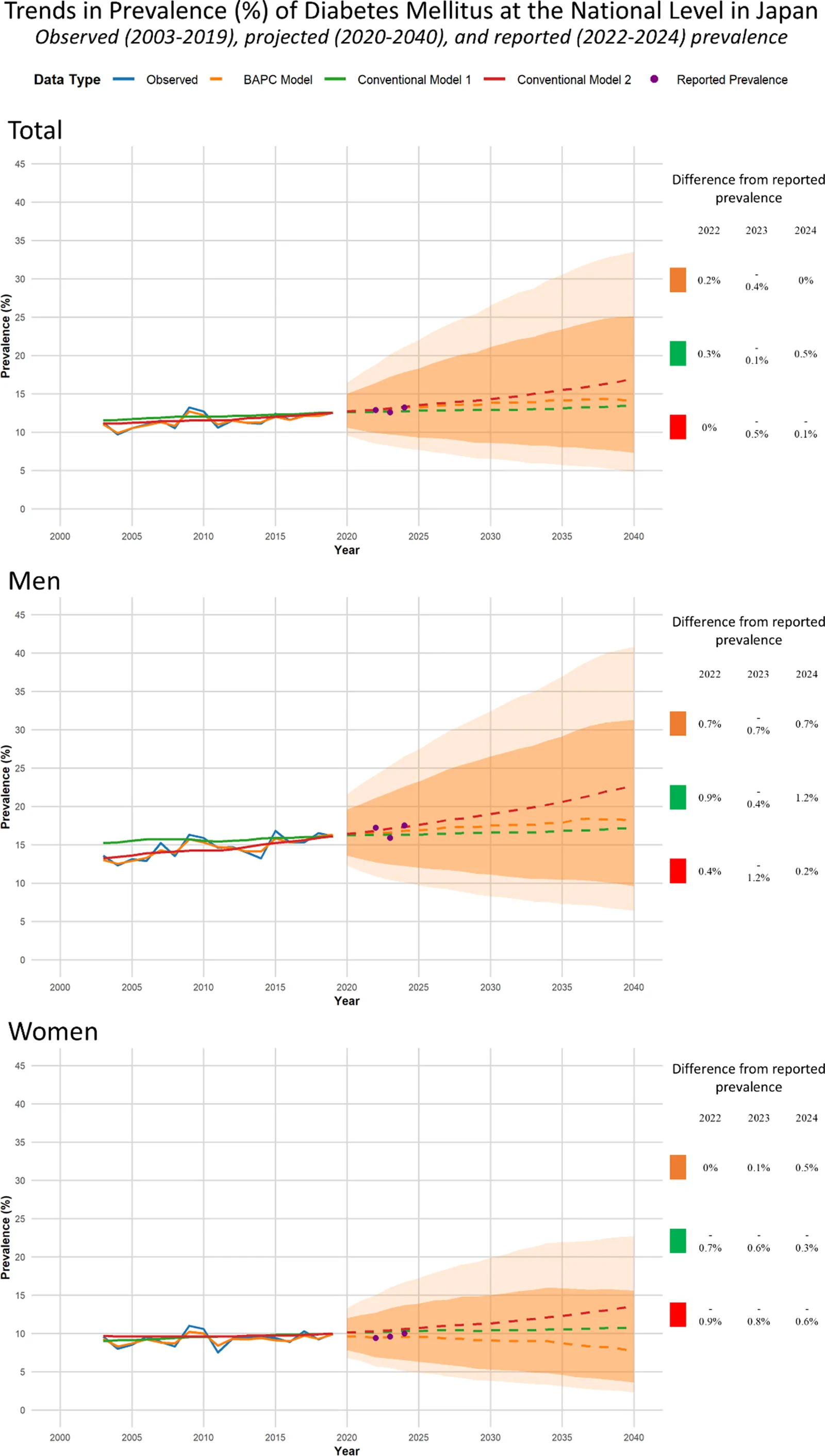
Illustrates trends in the crude prevalence (%) of diabetes mellitus at the national level in Japan observed from 2003 to 2019 and projected from 2020 to 2040 using the BAPC model and conventional models, aligned with the reported observed prevalence in 2022 and 2023. BAPC, Bayesian age-period-cohort; BAMP, Bayesian age-period-cohort modeling and prediction; and MCMC, Markov Chain Monte Carlo. BAPC model: The BAPC modeling with the R *bamp* package, where the future prevalence of diabetes is projected considering the age, period, and cohort effects using the random walks of first and second order (RW1 or RW2). The medians of their MCMC samples were used as the projection results and are presented in orange dashed lines (median) and shadings (outer light band: 95% credible intervals; inner darker band: 80% credible intervals). Projected values derived from Bayesian predictions differ from estimates directly derived from observed data. Credible intervals depict posterior predictive uncertainty and are not directly comparable with frequentist confidence intervals for observed estimates (such as mean, prevalence, odds ratio, hazard ratio, etc.). Projection credible intervals are usually wider than those for estimates based on observed data as the forecast horizon increases because uncertainty increases over future periods. Alongside 95% credible intervals, we reported the 80% credible intervals to represent the more concentrated central region of the MCMC posterior predictive distribution, which reflects lower probability coverage. Conventional model 1: The conventional prediction model with constant prevalence of diabetes, where the future prevalence is projected assuming a constant prevalence as observed in the last year of observed data (2019 in this study). Their projection results are presented in green dashed lines. Conventional model 2: The conventional prediction model with monotonic changes in diabetes prevalence, where the future prevalence is projected by using the prevalence that monotonically changed according to the mean percentage changes observed from 2003 to 2019. Their projection results are presented in red dashed lines. Reported prevalence: The reported prevalence refers to the prevalence of diabetes across 2022–2024, as presented in the National Health and Nutrition Survey report. These data are presented in dot points. The closer the dots are to the projection lines for each model, the better the predictive performance of the corresponding projection models. Age-specific observed diabetesprevalence among NHNS participants from 2003 to 2019 and national-level age-specific population projections from 2020 to 2040 in Japan were modeled in the BAPC to generate national-level age-specific and age-aggregated overall diabetes projections. Age-specific projected prevalence data from the BAPC were then applied to national-level population projections data to estimate the age-aggregated prevalence at the national level. The definition of diabetes corresponded to the official NHNS's ‘strongly suspected diabetes’ category (ascertained by HbA1c% ≥6.5, or medication use), rather than strictly diagnosed diabetes.

### RMSE comparison and model validation

Table 2 compares the RMSE values of the BAPC model with those of two conventional models for the validation dataset from 2015 to 2019. We finally adopted the BAPC model with RW2 for age effects and RW1 for period and cohort effects for total and men, and RW1 for age, period, and cohort effects for women, without heterogeneity, yielding the lowest RMSE value across 16 BAPC model structures by total and both sexes. The BAPC model demonstrated the lowest RMSE value (the smallest difference between observed and projected prevalences), considering both total and sex-specific values, highlighting the best predictive accuracy and reliability for our research.

**Table 2:** Prediction performance by RMSE values in the validation dataset from 2015 to 2019, derived from the BAPC model and conventional predicting models developed in the training dataset from 2003 to 2014, in the NHNS participants, Japan.

Population	RMSE		
	BAPC model	Conventional model 1	Conventional model 2
Total	26.7	32.2	21.2
Men	40.1	51.3	42.4
Women	11.5	20.8	23.1

RMSE, root mean square error; BAPC, Bayesian age-period-cohort; NHNS, National Health and Nutrition Survey; and BAMP, Bayesian age-period-cohort modeling and prediction.

BAPC model: The BAPC modeling with the R *bamp* package, where the future prevalence of diabetes is projected considering the age, period, and cohort effects using the random walks of first and second order (RW1 or RW2).

Conventional model 1: The conventional prediction model with constant prevalence of diabetes, where the future prevalence is projected assuming a constant prevalence as observed in the last year of observed data (2019 in this study).

Conventional model 2: The conventional prediction model with monotonic changes in diabetes prevalence, where the future prevalence is projected by using the prevalence that monotonically changed according to the mean percentage changes observed from 2003 to 2019.

RMSE shows the annual average error in the total count of cases (absolute numbers) across 2015–2019 in NHNS participants.

### DM prevalence and case projections by the BAPC model: main results

The projected median DM prevalence obtained from the BAPC model is plotted in Fig. 1. The detailed projection trends are shown in Table 3, by sex and age groups. The BAPC model indicated that median (95% CI [i.e., 2.5% and 97.5%]) DM prevalence was projected to increase from 12.6% (9.6%–16.4%) in 2020 to 14.0% (4.8%–33.5%) in 2040. While the prevalence in men was projected to increase from 16.4% (12.3%–21.6%) to 18.1% (6.4%–40.8%), the prevalence in women was projected to decline from 9.6% (6.8%–13.3%) to 7.7% (2.3%–22.7%) (Table 3 and Supplementary Table S4). Increasing trends were particularly notable among men aged 70 years and older, whereas decreasing trends were observed across all age groups in women (Table 3).

**Table 3:** Trends in age-specific and age-aggregated projected crude prevalence (%) of diabetes mellitus at the national level estimated using the BAPC model, Japan, from 2020 to 2040.

Population	2020	2030	2040
	Median (95% credible intervals)		
Total			
40–44 years	2.6 (1.6–4.0)	2.5 (0.9–6.9)	2.5 (0.6–9.6)
45–49 years	4.1 (2.8–5.8)	3.8 (1.5–9.2)	3.8 (1.0–13.0)
50–54 years	6.9 (4.9–9.5)	5.6 (2.4–12.8)	5.5 (1.7–16.9)
55–59 years	9.6 (7.1–12.9)	9.1 (4.0–19.4)	7.5 (2.3–21.5)
60–64 years	12.2 (9.1–16.0)	12.3 (5.6–25.1)	11.2 (3.7–29.9)
65–69 years	17.8 (13.7–22.8)	16.4 (7.6–31.5)	15.8 (5.4–38.9)
70–74 years	19.8 (15.4–25.2)	20.2 (9.7–37.4)	19.2 (6.7–44.3)
75–79 years	20.2 (15.6–25.6)	22.4 (10.9–40.6)	22.5 (8.1–49.5)
≥80 years	20.0 (15.6–25.5)	23.4 (11.5–42.3)	23.7 (8.6–51.3)
Overall	12.6 (9.6–16.4)	13.8 (6.5–26.5)	14.0 (4.8–33.5)
Men			
40–44 years	3.7 (2.1–6.0)	3.7 (1.3–9.9)	3.6 (0.9–13.5)
45–49 years	7.1 (4.8–10.4)	5.9 (2.3–13.7)	5.9 (1.6–19.0)
50–54 years	11.1 (7.8–15.5)	10.2 (4.4–21.9)	8.9 (2.7–25.8)
55–59 years	15.5 (11.3–20.8)	15.1 (6.8–29.9)	13.6 (4.3–34.9)
60–64 years	17.6 (13.1–23.3)	18.2 (8.5–34.6)	17.4 (5.9–41.9)
65–69 years	24.4 (18.9–30.9)	23.7 (11.5–42.1)	23.6 (8.5–50.9)
70–74 years	24.7 (19.1–31.3)	27.3 (13.7–46.8)	26.7 (9.8–55.1)
75–79 years	24.4 (18.7–30.9)	26.1 (13.0–45.1)	29.1 (11.0–58.1)
≥80 years	23.9 (18.2–30.6)	25.6 (12.6–44.7)	26.2 (9.7–54.3)
Overall	16.4 (12.3–21.6)	17.5 (8.3–32.4)	18.1 (6.4–40.8)
Women			
40–44 years	2.2 (1.2–3.8)	2.2 (0.6–7.2)	2.2 (0.4–10.3)
45–49 years	2.3 (1.4–3.6)	2.4 (0.8–6.6)	2.4 (0.5–10.1)
50–54 years	4.2 (2.8–6.3)	2.5 (0.9–6.5)	2.7 (0.7–10.0)
55–59 years	5.5 (3.7–8.2)	4.1 (1.6–10.2)	2.6 (0.7–9.0)
60–64 years	8.2 (5.7–11.6)	6.2 (2.5–14.9)	4.4 (1.2–14.6)
65–69 years	11.9 (8.5–16.3)	8.5 (3.5–19.6)	6.4 (1.8–20.4)
70–74 years	15.1 (11.0–20.3)	11.3 (4.7–24.9)	8.5 (2.5–25.6)
75–79 years	16.6 (12.1–22.3)	15.6 (6.8–32.2)	11.2 (3.4–31.7)
≥80 years	16.2 (11.8–22.0)	17.6 (7.7–35.5)	15.2 (4.8–40.0)
Overall	9.6 (6.8–13.3)	9.1 (3.8–19.9)	7.7 (2.3–22.7)

NHNS, National Health and Nutrition Survey; BAPC, Bayesian age-period-cohort; and BAMP, Bayesian age-period-cohort modeling and prediction.

Age-specific observed diabetes prevalence among NHNS participants from 2003 to 2019 and national-level age-specific population projections from 2020 to 2040 in Japan were modeled in the BAPC to generate national-level age-specific diabetes projections. Age-specific prevalence data from the BAPC were then applied to national-level population data to estimate the age-aggregated prevalence at the national level.

BAPC model: The BAPC modeling with the R *bamp* package, where the future prevalence of diabetes is projected considering the age, period, and cohort effects using the random walks of first and second order (RW1 or RW2).

The definition of diabetes corresponded to the official NHNS's ‘strongly suspected diabetes’ category (ascertained by HbA1c% ≥6.5, or medication use), rather than strictly diagnosed diabetes.

The median (95% CI) of DM case numbers (×100,000) was projected to increase from 99.2 (75.3–129.1) in 2020 to 103.9 (36.0–249.2) in 2040. While cases (×100,000) in men were projected to increase from 60.7 (45.3–79.8) to 63.4 (22.3–142.9), in women were projected to decline from 40.0 (28.4–55.4) to 30.3 (9.1–89.1) (Supplementary Table S4).

### DM prevalence and case projections by the conventional models

The conventional model 1 projected DM prevalence to increase from 12.6% (men, 16.2%; women, 10.1%) in 2020 to 13.5% (men, 17.2%; women, 10.8%) in 2040. The conventional model 2 projected it to increase from 12.7% (men, 16.4%; women, 10.1%) in 2020 to 17.0% (men, 22.7%; women, 13.6%) in 2040 (Fig. 1).

With the lowest RMSE values (Supplementary Table S5), BAPC-projected prevalences were close to the NHNS-reported prevalence in 2022–2024, compared with those from conventional models (Fig. 1).

### Age, period, and cohort effects

Coefficients for age, period, and cohort effects from the BAPC were estimated to interpret their corresponding effects on changes in the projected diabetes prevalence trends represented in Fig. 1. The estimated age-effect (Supplementary Figure S1) and period-effect (Supplementary Figure S2) patterns mainly indicated increasing trends in diabetes prevalence in the total population and men. The estimated age-effect and cohort-effect (Supplementary Figure S3) patterns, followed by the period-effect patterns, indicated sex-specific variations in diabetes prevalence trends. The estimated age-effect pattern in men suggested an upward curvature until age 65–69 years, followed by a modest increase thereafter. The cohort-effect pattern in men suggested modest upward curvature in older and intermediate birth cohorts, then downward curvature in the younger cohort. With an initially higher estimated cohort-effect pattern in women, it suggested modest downward curvature in the older birth cohort and eventually displayed downward curvature in intermediate and younger cohorts.

The age-standardized DM prevalence was projected to remain relatively stable for total (12.6% [9.6%–16.4%]–12.4% [4.2%–30.6%]) and men (17.0% [12.7%–22.3%]–17.2% [6.0%–39.4%]) and decrease in women (9.1% [6.4%–12.7%]–6.2% [1.8%–19.1%]) from 2020 to 2040 (Supplementary Table S6). Decomposition analysis revealed that the increasing DM prevalence in men was attributed to rising age-specific prevalence and population aging over time, with the latter contributing more (Supplementary Figure S4).

Sensitivity analyses yielded consistent results with our main findings. In the 1st sensitivity analysis, DM prevalence was projected to increase from 16.4% (12.2%–21.6%) in 2020 to 17.9% (6.3%–41.1%) in 2040 in men, while it was projected to decline from 9.7% (6.9%–13.5%) to 8.2% (2.3%–23.9%) in women (Supplementary Table S7). There were no notable differences in projected DM prevalence between the 2nd sensitivity analysis and the main analysis (Supplementary Table S8).

## Discussion

### Perspective and significance

This study estimated the future prevalence of DM in Japan using BAPC modeling that adjusts for age, period, and cohort effects, which are essential components for accurate DM projections. Therefore, the present study may provide a more robust and valid projected trend of DM prevalence and case numbers. To the best of our knowledge, this is the first study, particularly in this region, to comprehensively report sex-specific future projections in DM prevalence trends using BAPC modeling with detailed age, period, and cohort effects. Our study projected that the number of DM cases and prevalence to increase from 2020 to 2040 in Japan. While this trend is projected to increase in men, it is expected to decline in women. Crude prevalence of diabetes in men was projected to increase, though its age-standardized prevalence was projected to be flat. Our study highlights the increasing future burden of DM in a representative Asian nation.

### Our study findings compared to others

Prior studies mainly relied on traditional prediction modeling to project DM prevalence, including linear modeling used globally and regionally,^25^ dynamic Markov modeling used in the United States,^26^ Bayesian meta-regression modeling used globally, regionally, and in Japan (the GBD study),^2^ Bayesian time-series modeling used in China,^27^ and Bayesian inference with individual-based modeling used in Singapore.^28^ Consequently, our estimates differ from those of the above studies. The GBD study projected that the age-standardized total DM prevalence to increase from 6.1% in 2021 to 9.8% in 2050 globally among people aged 20 years and older, and from 5.9% in 2021 to 6.7% in 2050 in Japan. Our BAPC model projected that the age-standardized DM prevalence among people aged 40 years and older would remain relatively stable for total (12.6%–12.4%) and men (17.0%–17.2%) and decrease in women (9.1%–6.2%) from 2020 to 2040. We acknowledge that a direct comparison of our findings with those of the GBD study may not be appropriate, given differences in denominators and DM-ascertainment methods. In the GBD study, DM was defined as casual serum glucose in Japan until 2002, followed by HbA1c later; indirect estimation of type 2 DM prevalence as total DM minus type 1 DM prevalence; and a denominator of all age groups (20 years and older for the GBD whereas 40 years old and older for the present study), which were subsequently used for age-standardization.^2^ The Health Japan-21 project forecasted the prevalence of suspected diabetes to rise by age: 1.42% (20–39 years), 6.89% (40–49 years), 20.53% (50–59 years), 28.86% (60–69 years), and 31.82% (70 years and above) in men; and 1.29%, 2.61%, 4.27%, 9.88%, and 24.90% across the same age groups in women, by 2032.^7^ Despite differences in age groups, these sex-specific and age-specific prevalences are higher than our forecasted prevalence. However, the Health Japan-21 project's forecast of the prevalence of suspected diabetes (HbA1c% ≥6.5 or medication use) was limited to the short-term projection until 2032. The International Diabetes Federation Diabetes Atlas, 9th edition, estimated DM prevalence to be 11.4% in 2019, projected to rise to 12.4% by 2030 and 12.8% by 2045 in adults aged 20–79 years in the Western Pacific region.^25^ A recent study using a calibrated system dynamics simulation model projected that diabetes cases were expected to increase from 5.32 million in 2015 to 5.55 million in 2035 in men, and expected to decrease from 3.39 million to 3.29 million in women.^29^ This projected number is lower than our results. Our BAPC-projected prevalences in 2022–2024 were close to the NHNS-reported prevalence than those of conventional models, particularly in women (Fig. 1).^16^, ^17^, ^18^

A Chinese study projected this prevalence to increase from 8.2% in 2020 to 9.7% in 2030 in its population, using Bayesian time-series modeling.^27^ This prevalence was projected to double (from 7.3% to 15%) among Singaporean adults aged 18–69 from 1990 to 2050.^28^ While most studies projected an increase in DM prevalence, our study projected an increase in men but a slight decrease in women. However, we acknowledge that wider credible intervals may reflect greater uncertainty in long-term projections.

Another notable differentiating factor in our study was the inclusion of the middle-aged and older population. Therefore, despite the reported decrease in serum glucose levels in Japan from 2003 to 2018,^8^ we found increasing trends in DM prevalence over time. Data suggest that the consultation rate for diabetes is increasing in Japan.^30^ This could result in controlled serum glucose levels in recent years through lifestyle modification or medication use. This might also result in a higher number of diagnosed DM cases and medication use in recent years. This higher prevalence may be attributable to increasing unhealthy lifestyle factors (i.e., decreased dietary fiber intake and physical activity)^21^^,^^22^ and to higher body mass index^10^ over the last decade.

We recognized that the projected DM prevalence trend was increasing in men but declining in women. This discrepancy may be related to higher identifiable nonlinear estimated age-effect patterns more pronounced among men and lower identifiable nonlinear estimated cohort-effect patterns more pronounced among women. Historically, DM prevalence was higher in men from 2003 to 2019. Despite an initially lower estimated age-effect pattern in men in the very first age group, this pattern suggested an upward curvature until the 65–69 years age group, followed by a modest upward curvature thereafter, exceeding that of women. The estimated age-effect pattern and cohort-effect pattern suggested a more predominant role in reflecting DM trends than the period-effect pattern. Higher estimated age-effect pattern in increasing DM trends can be attributed to aging-related biological changes and deterioration in glucose metabolism.^31^^,^^32^ Population aging could be another notable contributing factor for higher DM prevalence. Owing to population aging in Japan, more people are moving into older age groups with higher diabetes prevalence, which is aligned with our decomposition analysis findings. An increasing estimated period-effect pattern may be attributed to growing access to healthcare, mass screening, and consultation rates.^30^

Despite a relatively lower estimated cohort-effect pattern in men in older birth cohorts than in women, this trend suggested a modest upward curvature in later cohorts and eventually surpassed that in women in the younger cohorts. Conversely, the estimated cohort-effect pattern displayed downward curvature in the intermediate and younger cohorts. This pattern suggests that DM prevalence gradually increased across all generations in men, while it declined steeply in the intermediate and younger generations in women. Nutritional deficiencies in early life are likely to trigger the high incidence of DM in adulthood.^33^ It is worth noting that the social unrest in Japan during World War II and its post-war effects may have contributed to poor early-life nutrition among the population cohort born between 1940 and 1955, which represents the intermediate birth cohort in our analysis. Later, advancements in healthcare may help stabilize or reduce the prevalence of DM, especially in women. However, an unhealthy lifestyle^16^^,^^17^ and higher body mass index^8^ in Japan over the last decades might drive higher DM rates, mainly among men. The trend in the prevalence of overweight/obesity, which is closely linked to DM, has been reported to be notably higher in men than in women in this country, suggesting influenced by the cohort-effect pattern. These findings support our results and further justify considering the use of the BAPC model in our study.^34^

We acknowledge that the narrower 80% credible intervals presented in the Fig. 1 do not indicate reduced underlying uncertainty. Rather, it represents the more concentrated central region of the posterior predictive distribution, reflects lower probability coverage, and is presented alongside 95% credible intervals.

### Policy implications

Our study has significant public health implications for policy reformation. Our data suggest that Japan may face a significant public health, societal, and economic burden due to soaring DM among its population. This concern is supported by previous modeling studies in Japan, which showed that increases in DM prevalence partly offset reductions in CHD deaths attributable to medical and surgical treatments and favorable changes in CVD risk factors between 1980 and 2012, while changes in HbA1c levels between 2001 and 2019 had only a limited impact on reductions in CHD and stroke incidence, all-cause deaths, direct and indirect costs, and gains in quality-adjusted life years.^35^^,^^36^ It is necessary to reform and implement effective public health policies to tackle the future burden of DM. This may include strengthening the healthcare system for screening and early detection of DM in primary care, meeting health needs, especially for older adults and men, monitoring the burden of DM, taking measures to prevent DM and its complications (e.g., lifestyle interventions), and other relevant measures.

### Strengths and limitations

Highlighting our strengths, we first used nationally representative samples and employed meticulous, complex random sampling methods. Second, we employed advanced, robust BAPC models using BAMP, which incorporates age, period, and cohort components and accounts for their effects. Third, we compared the predictive performance of our models using the RMSE of absolute differences. Fourth, we comprehensively compared our BAPC model's projection results with those of other conventional models.

However, some limitations should be discussed. First, we could not adjust our predictive models to account for other factors, such as socioeconomic factors, lifestyle factors, and body mass index, to consider their potential effects on DM projections. Second, the possibility of underreporting or overreporting DM due to a one-time glycemic status measurement cannot be entirely ruled out. We further acknowledge that relying on “strongly suspected diabetes” and omitting consideration of the “diabetes cannot be ruled out” category might potentially underestimate the prevalence of diabetes in our analysis. Third, reliance on HbA1c data, available since 2003, limited our ability to estimate historical DM trend data over 17 years, which may not be long enough for projection modeling over a longer period.

### Conclusions

The Bayesian age-period-cohort model demonstrated greater predictive accuracy in forecasting future trends in diabetes prevalence in Japan than other traditional models. This study suggests that DM prevalence at the national level in Japan is projected to increase from 16.4% (12.3%–21.6%) to 18.1% (6.4%–40.8%) in men between 2020 and 2040, while in women it is projected to decline from 9.6% (6.8%–13.3%) to 7.7% (2.3%–22.7%), mainly suggested by population age effects and cohort effects. Our study highlights the urgency of reforming and implementing effective public health policies to challenge the potential future burden of diabetes in Japan.

## Contributors

Conception and design: RM, SO, YY, and KN; acquisition of data: RM, SO, and YY; directly accessed and verified the data: RM and SO; analysis and interpretation of data: RM and SO; drafting the manuscript: RM; revising the manuscript critically for important intellectual content: all authors; supervision: SO and KN; final approval of the version to be published: all authors.

## Data sharing statement

The National Health and Nutrition Survey data are accessible from the Ministry of Health, Labour and Welfare, Japan, through a standard procedure.

## Declaration of interests

None.
