# Supplementary material for: Projections of diabetes mellitus in Japan through 2040: a bayesian age-period-cohort analysis of the national health and nutrition survey population
Source: Lancet Reg Health West Pac. 2026 Sep 22;74:101988. doi: 10.1016/j.lanwpc.2026.101988 (PMC13631670; doi:10.1016/j.lanwpc.2026.101988)
Supplement: Supplementary file 1 [file mmc1.docx]

**Supplemental Material**

**Projections of diabetes mellitus in Japan through 2040: a Bayesian age-period-cohort analysis of** **the National Health and Nutrition Survey population**

Rajib Mondal, MPH, PhD^1,2^, Soshiro Ogata, PhD^1^, Eri Kiyoshige, PhD^1^, Yuriko Nakaoku, MD, PhD^1^, Kiyomasa Nakatsuka, PhD^1^, Tomomi Watanabe, MD^1^, Kanako Teramoto, MD, PhD, MPH^3^, Aya Kadota, MD, PhD^2^, Katsuyuki Miura, MD, PhD^2^, Chris Kypridemos, MD, MPH, PhD^4^, Yusuke Yoshikawa, MD, PhD, MPH^1,5^, Kunihiro Nishimura, MD, PhD^1,3^

1 Department of Preventive Medicine and Epidemiology, National Cerebral and Cardiovascular Center, Osaka, Japan.

2 Department of Preventive Medicine, Shiga University of Medical Science, Shiga, Japan.

3 Department of Biostatistics, National Cerebral and Cardiovascular Center, Osaka, Japan.

4 Department of Public Health, Policy and Systems, University of Liverpool, Liverpool, United Kingdom.

5 Department of Data Science, Hyogo Medical University, Hyogo, Japan.

**Supplementary Methods:**

**BAPC statistical modeling:**

**a. BAPC statistical modeling using BAMP:** We developed BAPC modeling employing the R *bamp* package, fitting the population and DM case data stratified by sex and 9 age groups (40-44, 45-49, 50-54, 55-59, 60-64, 65-69, 70-74, 75-79, and ≥80 years) in a Lexis diagram. A Lexis diagram represents a two-dimensional data presentation tailored for a specific age group and a specific time. In each pixel of the Lexis diagram, data must be available on the number of people at risk (population) and the number of disease or mortality cases (incidence or mortality). The BAMP assumes a hierarchical model with a binomial model for event counts or number of cases, given the population at risk at the first stage, followed by assigning smoothing priors for APC parameters using the random walks of first and second order (RW1 or RW2) [1].

**b. BAPC model:** We used the BAPC model to estimate the projected prevalence of DM in the Japanese population from 2020 to 2040, using the historical observed DM prevalence data from 2003 to 2019. Practically, a similar approach was previously applied for projections of cardiovascular disease mortality in the United Kingdom [2], the United States [3], and Japan [4].

The BAPC model assumes the logit risk of incidence or event (i.e., mortality) in a specific age group and period follows a linear combination of an intercept and the APC parameter effects. In addition, APC effects can be constant or linear time trends (i.e., RW1 or RW2), with or without heterogeneity, which allowed us to consider various model structures. Hence, we identified the best model structure by calculating RMSE for both the total and sex-specific analyses. We split the dataset into a training dataset (2003-2014) and a validation dataset (2015-2019). We fitted models with all possible structures (16 patterns) to the validation dataset for both the total and the two sexes (i.e., a total of 48 models) and evaluated their predictive ability using RMSE on the validation dataset. We selected the best model structure with the lowest RMSE for the total and for each sex, and refitted it to the complete dataset covering 2003 to 2019 for our projections from 2020 to 2040. We compared the projected values from the BAPC models with the observed values from 2003 to 2019, both overall and by sex, as a model validation. We reported the age-specific projected crude prevalence of DM in median with 95% credible intervals (CI) (i.e., 2.5^th^ and 97.5^th^ percentiles) derived from the Markov chain Monte Carlo (MCMC) posterior distributions of the BAPC models.

**c. BAPC model structure:** We modeled the logit of the probability of prevalent diabetes mellitus in age group *i* in period *j* as a linear combination of an intercept $\mu$, age effect$\theta_{i}\left( i=1,\ldots I \right)$, period effect $\varphi_{j}\left( j=1,\ldots J \right)$ and the cohort effect$\psi_{k}\left( k=1,\ldots K \right)$:

$log\left( \frac{p_{ij}}{1-p_{ij}} \right)=\mu+\theta_{i}+\varphi_{j}+\psi_{k}.$

$I$ is the number of age groups, while $J$ is the number of periods. Cohorts are defined by $k=C\left( I-i \right)+j$ and the number of cohorts is $K=C\left( I-1 \right)+J$, where $C$ is the width of the age groups. To make the intercept $\mu$ identifiable, so-called “sum-to-zero” constraints must be imposed.

The term ‘identifiable’ referred to as well as indicated to the intercept (μ) parameter of the BAPC model, which is identifiable and estimated together with the age, period, and cohort effects. In a BAPC model, the intercept (μ) represents the overall average log rate through centering the age, period, and cohort effects using sum-to-zero constraints; in other words, the average of each age, period, and cohort effect is forced to be zero. Consequently, the intercept is uniquely identifiable after centering the age, period, and cohort effects.

In contrast, the individual linear age, period, and cohort effects are inherently non-identifiable in BAPC because of their exact linear dependency, as follows: cohort = period - age. It indicates that the overall linear effects in the BAPC model cannot be uniquely attributed to any one of the three components' temporal dimensions. This is a non-identifiable issue in BAPC, meaning that the APC’s nonlinear effects (curvatures) are only identifiable, but their linear effects (slopes) are non-identifiable.

**d. Random walk of first and second order (RW1 and RW2) in BAPC models:** In the BAPC model, the APC parameter ($\theta,\varphi, and \psi$) effects can be a constant trend over the time scale or linear time trend (i.e., referred to as random walks of first [RW1] or second order [RW2], respectively) with or without heterogeneity. Two types of priors describe such a relationship: first-difference and second-difference priors.

In the first-difference prior, APC effects are derived from the immediately preceding effect, which makes them closer to adjacent estimates (preventing large variation). Furthermore, a restriction is added to bound the first differences to zero stochastically, thereby preserving a constant trend. This refers to RW1. The age effect is explained by:

$\theta_{i}\sim N\left( \theta_{i-1},\kappa^{-1} \right), i=2,\ldots,I$.

$$p(\theta_{1})\propto const.$$

Where the hyperparameter $\kappa$ is also known as the precision parameter, in which larger values allow baseline effects to vary only slightly, while smaller values allow for more significant variation.

In the second difference prior, APC effects are yielded from its two immediate predecessors. This is known as RW2. This RW2 restricts the second differences stochastically to zero, which penalizes deviations from a linear trend:

$\theta_{i}\sim N\left( 2\theta_{i-1}-\theta_{i-2},\kappa^{-1} \right), i=2,\ldots,I$.

$$p(\theta_{1})=p(\theta_{2})\propto const.$$

In both prior types, the hyperparameter $\kappa$ is assumed to follow a gamma distribution $G\left( a,b \right).$ For RW1 and RW2 of the age effect, $a=1$ and $b=0.5$are set as initial values. For RW1 and RW2 of the period effect $\varphi$ and the cohort effect $\psi$, respectively, with hyperparameters $\lambda$ and $\nu$, $a=0$ and $b=0.00005$ are set as initial values.

Non-identifiability issues can arise in RW2 of the APC models. The RW2 parameters are unidentifiable since RW2 assumes a linear trend on the time scale. RW1 addresses this issue by keeping APC effects as constant as possible, since non-identifiability only affects linear trends [1]. Favorably, in the Bayesian framework, it is not essential to ensure identifiability of the APC effects, because $log\left( \frac{p_{ij}}{1-p_{ij}} \right)$ is fully identified [5]. In addition, many have considered the identifiability issue in the APC framework as unsolvable [6]. Also, the model can be easily extended to account for additional unstructured heterogeneity, which cannot be explained by the APC effects but by unknown and unobserved covariates:

$log\left( \frac{p_{ij}}{1-p_{ij}} \right) =\mu+\theta_{i}+\varphi_{j}+\psi_{k}+z_{ij}$.

$$z_{ij}\sim N\left( 0,\delta^{-1} \right),$$

where the hyperparameter $\delta$ is assumed to follow a gamma distribution$G\left( a_{\delta},b_{\delta} \right)$ with the same initial values as $\kappa.$

**e. Estimations of BAPC models and projection:** The models can be implemented in the *bamp* package of R statistical software, which uses MCMC simulation for the model estimation [1]. Once the model has converged to its stationary distribution, samples from the chain are used to calculate parameter estimates and credible intervals from the posterior distribution. A $100\left( 1-2p \right)\%$credible interval $\left[ c_{p}-c_{1-p} \right]$is a Bayesian interval estimate where $c_{p}$is equal to the $p^{th}$quantile of the scalar component and $c_{1-p}$is equal to the $\left( 1-p \right)^{th}$quantile.

In BAPC, future rates $p_{ij}, j=J+1,\ldots j+T$ can be calculated by projecting the APC effects into the future by repeating the application of the RW1 or RW2 in the model definitions. Similarly, projected effects $z_{ij}, j=J+1,\ldots j+T$ can be calculated independently of$N\left( 0,\delta^{-1} \right)$.

Note that the interpretation of credible intervals (Bayesian statistics) differs from that of frequentist confidence intervals (frequentist statistics). For example, a 95% credible interval of 15.50%-65.50% indicates that the posterior probability where a true percentage change lies between 15.50% and 65.50% is 95%. In this regard, credible intervals capture current uncertainty about the location of the true parameter value and thus can be interpreted as probabilistic statements about the parameter. On the other hand, confidence intervals capture the uncertainty about the interval obtained (i.e., whether it contains the true value). Thus, confidence intervals cannot be interpreted as a probabilistic statement about the true value of a parameter.

**MCMC posterior distributions settings:**

Our BAPC model fitting used MCMC with 300,000-600,000 iterations, 50% burn-in, thinning every 50 iterations, and 2,000 tuning iterations. This algorithm adaptation yielded approximately 3,000–6,000 posterior summaries and projections.

**Derivation of other national-level DM prevalence:**

We applied NHNS diabetes prevalence (medians in proportion) obtained from the BAPC model for the observed period and that of conventional model 1 and conventional model 2 for both observed and projected periods to the national-level population sizes from 2003 until 2040 (projected), to estimate the national-level observed and/or projected DM prevalence. First, we multiplied age-specific NHNS diabetes proportions by corresponding age-specific national population sizes for each year to estimate age-specific DM case numbers at the national level, both in total and by sex. Second, age-specific case numbers and population sizes were summed across age groups to obtain age-aggregated case numbers and population sizes. Third, we then divide age-aggregated case numbers by population sizes, multiplied by 100, to estimate overall age-aggregated DM prevalence at the national level for each year, both in total and by sex. We applied a similar strategy to NHNS-reported DM prevalence in 2022-2024 [21-23].

**Prior distributions in BAPC and sensitivity analysis:**

For random-walk components’ smoothing priors in the BAPC model, we specified Gamma priors on precision parameters (inverse of variance of the differences between adjacent effects) for each APC component as follows - $G\left( a,b \right)$. The higher parameters imply stronger smoothing. The hyperparameters $G\left( a,b \right)$ were derived assuming a plausible range of differences between adjacent levels, expressed in a log-scale increment of ±δ (delta). In our main analysis, we adopted random-walk smoothing priors with ±δ values of log(1.5) for all APC effects.

We conducted our sensitivity analysis using 2 strategies. First, we considered a set of random-walk smoothing priors, ranging from ±δ values of log(1.5) to log(5.0) in increments of 0.5, for each APC effect, individually. Finally, we specified prior smoothness with ±δ values of log(2.0) for the age effect, while keeping the priors unchanged for the period and cohort effects. Because other prior combinations yielded higher RMSE values (either in total or sex-specific estimates), a reduced number of converged models, and non-plausible projection estimates. Consequently, we omitted presenting their RMSE values and projection estimates. This prior setting tested whether our projection estimates and conclusions are robust. Second, NHNS’s expanded surveys in 2012 and 2016 were conducted with approximately 4 times the population size as in other usual survey cycles, which may unduly drive estimates of period and cohort effects. Also, NHNS survey weights were not considered in this current analysis. Therefore, we repeated the analysis with down-samples of one-quarter of the population and cases, deliberately dividing the age-specific population and case numbers by 4. We used the same prior ±δ value of log(1.5) as in the main analysis and additionally considered a set of Bayesian parameter combinations for down-samples. We further externally validate the projections by calculating RMSE compared with the official NHNS-reported DM prevalence across 2022-2024.

**Bayesian parameter settings and external validation of the down-samples’ results:**

We considered a set of Bayesian parameter-setting combinations, including full iterations and the tuning sizes used in the main analysis; half iterations and half the tuning sizes; half iterations and a quarter of the tuning sizes; and larger iterations and tuning sizes beyond those used in the main analysis. By calculating and comparing RMSE, we externally validated the projected DM prevalence results obtained from the BAPC model using down-samples and these iteration-tuning setting combinations, comparing them with the official NHNS-reported DM prevalence. We calculated validation RMSE as the annual average difference between BAPC-projected DM prevalence and the official NHNS-reported DM prevalence across 2022-2024, separately for the total and by each sex group and for each iteration-tuning combination. Thus, each iteration–tuning setting combination yielded its own RMSE for total, men, and women. Lower RMSE indicated better predictive performance, and the best model was thereby selected.

**Supplementary Tables**

**Supplementary Table 1**

| **Table S1: Number of observed populations among the nationally representative samples participating in the National Health and Nutrition Survey, Japan, from 2003 to 2019** | | | | | | | | | | | | | | | | | | |
| --- | --- | --- | --- | --- | --- | --- | --- | --- | --- | --- | --- | --- | --- | --- | --- | --- | --- | --- |
| **Population** | | **2003** | **2004** | **2005** | **2006** | **2007** | **2008** | **2009** | **2010** | **2011** | **2012** | **2013** | **2014** | **2015** | **2016** | **2017** | **2018** | **2019** |
| **Total** | |  |  |  |  |  |  |  |  |  |  |  |  |  |  |  |  |  |
|  | 40-44 years | 333 | 266 | 248 | 282 | 274 | 251 | 318 | 242 | 229 | 965 | 235 | 215 | 249 | 796 | 186 | 174 | 144 |
|  | 45-49 years | 409 | 253 | 257 | 288 | 283 | 250 | 296 | 241 | 202 | 870 | 190 | 222 | 228 | 736 | 196 | 201 | 182 |
|  | 50-54 years | 541 | 369 | 304 | 361 | 282 | 334 | 322 | 293 | 261 | 976 | 209 | 254 | 229 | 718 | 194 | 195 | 173 |
|  | 55-59 years | 524 | 400 | 407 | 514 | 429 | 458 | 421 | 356 | 281 | 1224 | 243 | 305 | 244 | 871 | 220 | 240 | 177 |
|  | 60-64 years | 579 | 491 | 434 | 435 | 508 | 549 | 507 | 494 | 454 | 1893 | 395 | 421 | 393 | 1248 | 318 | 282 | 215 |
|  | 65-69 years | 617 | 442 | 495 | 477 | 426 | 555 | 519 | 532 | 394 | 1737 | 459 | 504 | 497 | 1811 | 392 | 420 | 363 |
|  | 70-74 years | 521 | 394 | 448 | 441 | 422 | 526 | 447 | 436 | 428 | 1670 | 447 | 448 | 383 | 1335 | 404 | 387 | 360 |
|  | 75-79 years | 342 | 283 | 318 | 363 | 325 | 409 | 370 | 313 | 330 | 1273 | 337 | 347 | 320 | 1138 | 333 | 311 | 281 |
|  | ≥80 years | 291 | 186 | 233 | 248 | 261 | 344 | 310 | 288 | 315 | 1100 | 254 | 314 | 297 | 1159 | 296 | 302 | 247 |
|  | **Overall** | **4157** | **3084** | **3144** | **3409** | **3210** | **3676** | **3510** | **3195** | **2894** | **11708** | **2769** | **3030** | **2840** | **9812** | **2539** | **2512** | **2142** |
| **Men** | |  |  |  |  |  |  |  |  |  |  |  |  |  |  |  |  |  |
|  | 40-44 years | 118 | 81 | 79 | 99 | 100 | 93 | 125 | 95 | 79 | 346 | 83 | 78 | 83 | 306 | 57 | 69 | 48 |
|  | 45-49 years | 150 | 89 | 96 | 108 | 110 | 92 | 114 | 80 | 74 | 341 | 58 | 82 | 67 | 284 | 66 | 77 | 67 |
|  | 50-54 years | 208 | 133 | 104 | 142 | 104 | 130 | 120 | 122 | 102 | 359 | 84 | 98 | 78 | 266 | 71 | 67 | 59 |
|  | 55-59 years | 194 | 149 | 154 | 208 | 160 | 162 | 176 | 135 | 113 | 465 | 96 | 120 | 87 | 331 | 87 | 94 | 70 |
|  | 60-64 years | 248 | 211 | 190 | 182 | 217 | 226 | 214 | 231 | 190 | 818 | 177 | 176 | 162 | 509 | 138 | 118 | 84 |
|  | 65-69 years | 262 | 194 | 206 | 207 | 194 | 258 | 231 | 230 | 166 | 734 | 188 | 242 | 218 | 786 | 181 | 184 | 165 |
|  | 70-74 years | 238 | 181 | 223 | 207 | 197 | 241 | 193 | 192 | 193 | 730 | 215 | 201 | 187 | 589 | 177 | 165 | 164 |
|  | 75-79 years | 144 | 133 | 141 | 156 | 142 | 181 | 153 | 139 | 151 | 569 | 163 | 169 | 141 | 496 | 146 | 154 | 133 |
|  | ≥80 years | 114 | 80 | 86 | 109 | 107 | 151 | 117 | 129 | 138 | 465 | 116 | 144 | 134 | 488 | 134 | 120 | 107 |
|  | **Overall** | **1676** | **1251** | **1279** | **1418** | **1331** | **1534** | **1443** | **1353** | **1206** | **4827** | **1180** | **1310** | **1157** | **4055** | **1057** | **1048** | **897** |
| **Women** | |  |  |  |  |  |  |  |  |  |  |  |  |  |  |  |  |  |
|  | 40-44 years | 215 | 185 | 169 | 183 | 174 | 158 | 193 | 147 | 150 | 619 | 152 | 137 | 166 | 490 | 129 | 105 | 96 |
|  | 45-49 years | 259 | 164 | 161 | 180 | 173 | 158 | 182 | 161 | 128 | 529 | 132 | 140 | 161 | 452 | 130 | 124 | 115 |
|  | 50-54 years | 333 | 236 | 200 | 219 | 178 | 204 | 202 | 171 | 159 | 617 | 125 | 156 | 151 | 452 | 123 | 128 | 114 |
|  | 55-59 years | 330 | 251 | 253 | 306 | 269 | 296 | 245 | 221 | 168 | 759 | 147 | 185 | 157 | 540 | 133 | 146 | 107 |
|  | 60-64 years | 331 | 280 | 244 | 253 | 291 | 323 | 293 | 263 | 264 | 1075 | 218 | 245 | 231 | 739 | 180 | 164 | 131 |
|  | 65-69 years | 355 | 248 | 289 | 270 | 232 | 297 | 288 | 302 | 228 | 1003 | 271 | 262 | 279 | 1025 | 211 | 236 | 198 |
|  | 70-74 years | 283 | 213 | 225 | 234 | 225 | 285 | 254 | 244 | 235 | 940 | 232 | 247 | 196 | 746 | 227 | 222 | 196 |
|  | 75-79 years | 198 | 150 | 177 | 207 | 183 | 228 | 217 | 174 | 179 | 704 | 174 | 178 | 179 | 642 | 187 | 157 | 148 |
|  | ≥80 years | 177 | 106 | 147 | 139 | 154 | 193 | 193 | 159 | 177 | 635 | 138 | 170 | 163 | 671 | 162 | 182 | 140 |
|  | **Overall** | **2481** | **1833** | **1865** | **1991** | **1879** | **2142** | **2067** | **1842** | **1688** | **6881** | **1589** | **1720** | **1683** | **5757** | **1482** | **1464** | **1245** |
| NHNS’s expanded surveys were conducted in 2012 and 2016, corresponding to larger population sizes than in other survey cycles. | | | | | | | | | | | | | | | | | | |

**Supplementary Table 2**

| **Table S2: Number of observed diabetic cases among the nationally representative samples participating in the National Health and Nutrition Survey, Japan, from 2003 to 2019** | | | | | | | | | | | | | | | | | | |
| --- | --- | --- | --- | --- | --- | --- | --- | --- | --- | --- | --- | --- | --- | --- | --- | --- | --- | --- |
| **Population** | | **2003** | **2004** | **2005** | **2006** | **2007** | **2008** | **2009** | **2010** | **2011** | **2012** | **2013** | **2014** | **2015** | **2016** | **2017** | **2018** | **2019** |
| **Total** | |  |  |  |  |  |  |  |  |  |  |  |  |  |  |  |  |  |
|  | 40-44 years | 13 | 8 | 7 | 5 | 9 | 9 | 8 | 14 | 5 | 20 | 5 | 3 | 9 | 16 | 1 | 6 | 5 |
|  | 45-49 years | 22 | 14 | 9 | 11 | 17 | 9 | 25 | 11 | 7 | 41 | 7 | 7 | 6 | 25 | 13 | 11 | 7 |
|  | 50-54 years | 47 | 26 | 22 | 26 | 21 | 23 | 16 | 26 | 19 | 68 | 9 | 15 | 19 | 54 | 13 | 12 | 14 |
|  | 55-59 years | 49 | 41 | 38 | 58 | 33 | 45 | 63 | 33 | 21 | 109 | 23 | 27 | 28 | 80 | 20 | 23 | 17 |
|  | 60-64 years | 80 | 43 | 62 | 59 | 68 | 65 | 76 | 79 | 58 | 250 | 44 | 51 | 35 | 159 | 33 | 40 | 18 |
|  | 65-69 years | 99 | 72 | 91 | 66 | 89 | 82 | 101 | 87 | 69 | 281 | 71 | 80 | 91 | 301 | 62 | 79 | 67 |
|  | 70-74 years | 96 | 59 | 69 | 85 | 71 | 88 | 80 | 82 | 58 | 307 | 87 | 76 | 73 | 238 | 88 | 63 | 77 |
|  | 75-79 years | 65 | 39 | 49 | 58 | 55 | 58 | 78 | 66 | 59 | 217 | 60 | 69 | 66 | 203 | 77 | 64 | 64 |
|  | ≥80 years | 33 | 21 | 26 | 38 | 39 | 52 | 52 | 46 | 48 | 199 | 50 | 56 | 61 | 212 | 54 | 48 | 43 |
|  | **Overall** | **504** | **323** | **373** | **406** | **402** | **431** | **499** | **444** | **344** | **1492** | **356** | **384** | **388** | **1288** | **361** | **346** | **312** |
| **Men** | |  |  |  |  |  |  |  |  |  |  |  |  |  |  |  |  |  |
|  | 40-44 years | 6 | 1 | 1 | 3 | 6 | 5 | 6 | 11 | 4 | 11 | 4 | 2 | 3 | 9 | 0 | 2 | 2 |
|  | 45-49 years | 11 | 9 | 6 | 6 | 9 | 5 | 10 | 3 | 4 | 27 | 4 | 4 | 4 | 16 | 7 | 7 | 4 |
|  | 50-54 years | 27 | 14 | 11 | 13 | 14 | 9 | 7 | 16 | 13 | 37 | 4 | 8 | 9 | 34 | 9 | 9 | 7 |
|  | 55-59 years | 24 | 25 | 19 | 31 | 17 | 28 | 37 | 22 | 11 | 65 | 14 | 15 | 19 | 47 | 12 | 17 | 13 |
|  | 60-64 years | 40 | 16 | 37 | 29 | 36 | 36 | 39 | 46 | 32 | 152 | 27 | 29 | 22 | 95 | 17 | 21 | 8 |
|  | 65-69 years | 53 | 38 | 47 | 28 | 50 | 43 | 54 | 47 | 41 | 145 | 38 | 44 | 56 | 178 | 40 | 50 | 44 |
|  | 70-74 years | 48 | 40 | 41 | 50 | 45 | 46 | 42 | 42 | 36 | 160 | 52 | 41 | 42 | 120 | 48 | 38 | 46 |
|  | 75-79 years | 27 | 16 | 21 | 30 | 34 | 37 | 49 | 35 | 37 | 123 | 35 | 36 | 42 | 109 | 43 | 42 | 32 |
|  | ≥80 years | 18 | 12 | 15 | 18 | 18 | 30 | 21 | 19 | 30 | 98 | 23 | 30 | 34 | 120 | 23 | 18 | 22 |
|  | **Overall** | **254** | **171** | **198** | **208** | **229** | **239** | **265** | **241** | **208** | **818** | **201** | **209** | **231** | **728** | **199** | **204** | **178** |
| **Women** | |  |  |  |  |  |  |  |  |  |  |  |  |  |  |  |  |  |
|  | 40-44 years | 7 | 7 | 6 | 2 | 3 | 4 | 2 | 3 | 1 | 9 | 1 | 1 | 6 | 7 | 1 | 4 | 3 |
|  | 45-49 years | 11 | 5 | 3 | 5 | 8 | 4 | 15 | 8 | 3 | 14 | 3 | 3 | 2 | 9 | 6 | 4 | 3 |
|  | 50-54 years | 20 | 12 | 11 | 13 | 7 | 14 | 9 | 10 | 6 | 31 | 5 | 7 | 10 | 20 | 4 | 3 | 7 |
|  | 55-59 years | 25 | 16 | 19 | 27 | 16 | 17 | 26 | 11 | 10 | 44 | 9 | 12 | 9 | 33 | 8 | 6 | 4 |
|  | 60-64 years | 40 | 27 | 25 | 30 | 32 | 29 | 37 | 33 | 26 | 98 | 17 | 22 | 13 | 64 | 16 | 19 | 10 |
|  | 65-69 years | 46 | 34 | 44 | 38 | 39 | 39 | 47 | 40 | 28 | 136 | 33 | 36 | 35 | 123 | 22 | 29 | 23 |
|  | 70-74 years | 48 | 19 | 28 | 35 | 26 | 42 | 38 | 40 | 22 | 147 | 35 | 35 | 31 | 118 | 40 | 25 | 31 |
|  | 75-79 years | 38 | 23 | 28 | 28 | 21 | 21 | 29 | 31 | 22 | 94 | 25 | 33 | 24 | 94 | 34 | 22 | 32 |
|  | ≥80 years | 15 | 9 | 11 | 20 | 21 | 22 | 31 | 27 | 18 | 101 | 27 | 26 | 27 | 92 | 31 | 30 | 21 |
|  | **Overall** | **250** | **152** | **175** | **198** | **173** | **192** | **234** | **203** | **136** | **674** | **155** | **175** | **157** | **560** | **162** | **142** | **134** |
| NHNS’s expanded surveys were conducted in 2012 and 2016, corresponding to larger case numbers than in other survey cycles. | | | | | | | | | | | | | | | | | | |

**Supplementary Table 3**

| **Table S3: Trends in observed prevalence (%) of diabetes mellitus among the nationally representative samples participating in the National Health and Nutrition Survey, Japan, from 2003 to 2019** | | | | | | | | | | | | | | | | | | |
| --- | --- | --- | --- | --- | --- | --- | --- | --- | --- | --- | --- | --- | --- | --- | --- | --- | --- | --- |
| **Population** | | **2003** | **2004** | **2005** | **2006** | **2007** | **2008** | **2009** | **2010** | **2011** | **2012** | **2013** | **2014** | **2015** | **2016** | **2017** | **2018** | **2019** |
| **Total** | |  |  |  |  |  |  |  |  |  |  |  |  |  |  |  |  |  |
|  | 40-44 years | 3.9 | 3.0 | 2.8 | 1.8 | 3.3 | 3.6 | 2.5 | 5.8 | 2.2 | 2.1 | 2.1 | 1.4 | 3.6 | 2.0 | 0.5 | 3.4 | 3.5 |
|  | 45-49 years | 5.4 | 5.5 | 3.5 | 3.8 | 6.0 | 3.6 | 8.4 | 4.6 | 3.5 | 4.7 | 3.7 | 3.2 | 2.6 | 3.4 | 6.6 | 5.5 | 3.8 |
|  | 50-54 years | 8.7 | 7.0 | 7.2 | 7.2 | 7.4 | 6.9 | 5.0 | 8.9 | 7.3 | 7.0 | 4.3 | 5.9 | 8.3 | 7.5 | 6.7 | 6.2 | 8.1 |
|  | 55-59 years | 9.4 | 10.2 | 9.3 | 11.3 | 7.7 | 9.8 | 15.0 | 9.3 | 7.5 | 8.9 | 9.5 | 8.9 | 11.5 | 9.2 | 9.1 | 9.6 | 9.6 |
|  | 60-64 years | 13.8 | 8.8 | 14.3 | 13.6 | 13.4 | 11.8 | 15.0 | 16.0 | 12.8 | 13.2 | 11.1 | 12.1 | 8.9 | 12.7 | 10.4 | 14.2 | 8.4 |
|  | 65-69 years | 16.0 | 16.3 | 18.4 | 13.8 | 20.9 | 14.8 | 19.5 | 16.4 | 17.5 | 16.2 | 15.5 | 15.9 | 18.3 | 16.6 | 15.8 | 18.8 | 18.5 |
|  | 70-74 years | 18.4 | 15.0 | 15.4 | 19.3 | 16.8 | 16.7 | 17.9 | 18.8 | 13.6 | 18.4 | 19.5 | 17.0 | 19.1 | 17.8 | 21.8 | 16.3 | 21.4 |
|  | 75-79 years | 19.0 | 13.8 | 15.4 | 16.0 | 16.9 | 14.2 | 21.1 | 21.1 | 17.9 | 17.0 | 17.8 | 19.9 | 20.6 | 17.8 | 23.1 | 20.6 | 22.8 |
|  | ≥80 years | 11.3 | 11.3 | 11.2 | 15.3 | 14.9 | 15.1 | 16.8 | 16.0 | 15.2 | 18.1 | 19.7 | 17.8 | 20.5 | 18.3 | 18.2 | 15.9 | 17.4 |
|  | **Overall** | **12.1** | **10.5** | **11.9** | **11.9** | **12.5** | **11.7** | **14.2** | **13.9** | **11.9** | **12.7** | **12.9** | **12.7** | **13.7** | **13.1** | **14.2** | **13.8** | **14.6** |
| **Men** | |  |  |  |  |  |  |  |  |  |  |  |  |  |  |  |  |  |
|  | 40-44 years | 5.1 | 1.2 | 1.3 | 3.0 | 6.0 | 5.4 | 4.8 | 11.6 | 5.1 | 3.2 | 4.8 | 2.6 | 3.6 | 2.9 | 0.0 | 2.9 | 4.2 |
|  | 45-49 years | 7.3 | 10.1 | 6.2 | 5.6 | 8.2 | 5.4 | 8.8 | 3.8 | 5.4 | 7.9 | 6.9 | 4.9 | 6.0 | 5.6 | 10.6 | 9.1 | 6.0 |
|  | 50-54 years | 13.0 | 10.5 | 10.6 | 9.2 | 13.5 | 6.9 | 5.8 | 13.1 | 12.7 | 10.3 | 4.8 | 8.2 | 11.5 | 12.8 | 12.7 | 13.4 | 11.9 |
|  | 55-59 years | 12.4 | 16.8 | 12.3 | 14.9 | 10.6 | 17.3 | 21.0 | 16.3 | 9.7 | 14.0 | 14.6 | 12.5 | 21.8 | 14.2 | 13.8 | 18.1 | 18.6 |
|  | 60-64 years | 16.1 | 7.6 | 19.5 | 15.9 | 16.6 | 15.9 | 18.2 | 19.9 | 16.8 | 18.6 | 15.3 | 16.5 | 13.6 | 18.7 | 12.3 | 17.8 | 9.5 |
|  | 65-69 years | 20.2 | 19.6 | 22.8 | 13.5 | 25.8 | 16.7 | 23.4 | 20.4 | 24.7 | 19.8 | 20.2 | 18.2 | 25.7 | 22.6 | 22.1 | 27.2 | 26.7 |
|  | 70-74 years | 20.2 | 22.1 | 18.4 | 24.2 | 22.8 | 19.1 | 21.8 | 21.9 | 18.7 | 21.9 | 24.2 | 20.4 | 22.5 | 20.4 | 27.1 | 23.0 | 28.0 |
|  | 75-79 years | 18.8 | 12.0 | 14.9 | 19.2 | 23.9 | 20.4 | 32.0 | 25.2 | 24.5 | 21.6 | 21.5 | 21.3 | 29.8 | 22.0 | 29.5 | 27.3 | 24.1 |
|  | ≥80 years | 15.8 | 15.0 | 17.4 | 16.5 | 16.8 | 19.9 | 17.9 | 14.7 | 21.7 | 21.1 | 19.8 | 20.8 | 25.4 | 24.6 | 17.2 | 15.0 | 20.6 |
|  | **Overall** | **15.2** | **13.7** | **15.5** | **14.7** | **17.2** | **15.6** | **18.4** | **17.8** | **17.2** | **16.9** | **17.0** | **16.0** | **20.0** | **18.0** | **18.8** | **19.5** | **19.8** |
| **Women** | |  |  |  |  |  |  |  |  |  |  |  |  |  |  |  |  |  |
|  | 40-44 years | 3.3 | 3.8 | 3.6 | 1.1 | 1.7 | 2.5 | 1.0 | 2.0 | 0.7 | 1.5 | 0.7 | 0.7 | 3.6 | 1.4 | 0.8 | 3.8 | 3.1 |
|  | 45-49 years | 4.2 | 3.0 | 1.9 | 2.8 | 4.6 | 2.5 | 8.2 | 5.0 | 2.3 | 2.6 | 2.3 | 2.1 | 1.2 | 2.0 | 4.6 | 3.2 | 2.6 |
|  | 50-54 years | 6.0 | 5.1 | 5.5 | 5.9 | 3.9 | 6.9 | 4.5 | 5.8 | 3.8 | 5.0 | 4.0 | 4.5 | 6.6 | 4.4 | 3.3 | 2.3 | 6.1 |
|  | 55-59 years | 7.6 | 6.4 | 7.5 | 8.8 | 5.9 | 5.7 | 10.6 | 5.0 | 6.0 | 5.8 | 6.1 | 6.5 | 5.7 | 6.1 | 6.0 | 4.1 | 3.7 |
|  | 60-64 years | 12.1 | 9.6 | 10.2 | 11.9 | 11.0 | 9.0 | 12.6 | 12.5 | 9.8 | 9.1 | 7.8 | 9.0 | 5.6 | 8.7 | 8.9 | 11.6 | 7.6 |
|  | 65-69 years | 13.0 | 13.7 | 15.2 | 14.1 | 16.8 | 13.1 | 16.3 | 13.2 | 12.3 | 13.6 | 12.2 | 13.7 | 12.5 | 12.0 | 10.4 | 12.3 | 11.6 |
|  | 70-74 years | 17.0 | 8.9 | 12.4 | 15.0 | 11.6 | 14.7 | 15.0 | 16.4 | 9.4 | 15.6 | 15.1 | 14.2 | 15.8 | 15.8 | 17.6 | 11.3 | 15.8 |
|  | 75-79 years | 19.2 | 15.3 | 15.8 | 13.5 | 11.5 | 9.2 | 13.4 | 17.8 | 12.3 | 13.4 | 14.4 | 18.5 | 13.4 | 14.6 | 18.2 | 14.0 | 21.6 |
|  | ≥80 years | 8.5 | 8.5 | 7.5 | 14.4 | 13.6 | 11.4 | 16.1 | 17.0 | 10.2 | 15.9 | 19.6 | 15.3 | 16.6 | 13.7 | 19.1 | 16.5 | 15.0 |
|  | **Overall** | **10.1** | **8.3** | **9.4** | **9.9** | **9.2** | **9.0** | **11.3** | **11.0** | **8.1** | **9.8** | **9.8** | **10.2** | **9.3** | **9.7** | **10.9** | **9.7** | **10.8** |

**Supplementary Table 4**

| **Table S4. Trends in projected number of cases (×100,000) and prevalence (%) of diabetes mellitus at the national level derived from the BAPC model, Japan, from 2020 to 2040** | | | | | | | | | |
| --- | --- | --- | --- | --- | --- | --- | --- | --- | --- |
| **Year** | **Total** | | | **Men** | | | **Women** | | |
|  | **Population**  **(×100,000)** | **Case**  **(****×100,000)**  **Median (95% CI)** | **Prevalence**  **Median (95% CI)** | **Population**  **(×100,000)** | **Case**  **(×100,000)**  **Median (95% CI)** | **Prevalence**  **Median (95% CI)** | **Population**  **(×100,000)** | **Case**  **(×100,000)**  **Median (95% CI)** | **Prevalence**  **Median (95% CI)** |
| 2020 | 785.0 | 99.2 (75.3 - 129.1) | 12.6 (9.6 - 16.4) | 369.7 | 60.7 (45.3 - 79.8) | 16.4 (12.3 - 21.6) | 415.3 | 40.0 (28.4 - 55.4) | 9.6 (6.8 - 13.3) |
| 2021 | 785.9 | 99.8 (70.7 - 138.7) | 12.7 (9.0 - 17.7) | 370.2 | 60.9 (42.7 - 84.6) | 16.5 (11.5 - 22.9) | 415.7 | 39.9 (26.4 - 59.4) | 9.6 (6.4 - 14.3) |
| 2022 | 786.4 | 100.3 (66.5 - 147.9) | 12.7 (8.5 - 18.8) | 370.4 | 61.1 (40.2 - 89.4) | 16.5 (10.9 - 24.1) | 416.1 | 39.1 (23.9 - 62.3) | 9.4 (5.7 - 15.0) |
| 2023 | 786.7 | 102.3 (64.7 - 157.9) | 13.0 (8.2 - 20.1) | 370.5 | 61.5 (38.4 - 93.6) | 16.6 (10.4 - 25.3) | 416.3 | 39.7 (22.8 - 66.6) | 9.5 (5.5 - 16.0) |
| 2024 | 786.8 | 103.6 (62.2 - 166.2) | 13.2 (7.9 - 21.1) | 370.5 | 62.3 (37.4 - 98.3) | 16.8 (10.1 - 26.5) | 416.3 | 39.6 (21.4 - 69.3) | 9.5 (5.1 - 16.7) |
| 2025 | 786.3 | 103.7 (60.2 - 173.2) | 13.2 (7.7 - 22.0) | 370.2 | 62.5 (35.9 - 101.6) | 16.9 (9.7 - 27.4) | 416.1 | 39.3 (20.3 - 71.7) | 9.5 (4.9 - 17.2) |
| 2026 | 785.0 | 104.9 (58.1 - 181.2) | 13.4 (7.4 - 23.1) | 369.6 | 62.9 (34.8 - 105.5) | 17.0 (9.4 - 28.6) | 415.4 | 39.3 (19.4 - 74.2) | 9.5 (4.7 - 17.9) |
| 2027 | 783.4 | 105.7 (56.3 - 187.8) | 13.5 (7.2 - 24.0) | 368.8 | 63.7 (33.9 - 109.6) | 17.3 (9.2 - 29.7) | 414.6 | 38.7 (18.1 - 76.3) | 9.3 (4.4 - 18.4) |
| 2028 | 781.3 | 106.1 (54.0 - 193.8) | 13.6 (6.9 - 24.8) | 367.7 | 63.8 (32.7 - 112.7) | 17.3 (8.9 - 30.6) | 413.5 | 38.6 (17.4 - 78.4) | 9.3 (4.2 - 19.0) |
| 2029 | 778.9 | 105.3 (51.5 - 198.2) | 13.5 (6.6 - 25.4) | 366.6 | 63.6 (31.2 - 115.4) | 17.3 (8.5 - 31.5) | 412.3 | 37.4 (16.2 - 79.6) | 9.1 (3.9 - 19.3) |
| 2030 | 776.0 | 106.8 (50.3 - 205.8) | 13.8 (6.5 - 26.5) | 365.2 | 63.9 (30.3 - 118.5) | 17.5 (8.3 - 32.4) | 410.8 | 37.5 (15.7 - 81.7) | 9.1 (3.8 - 19.9) |
| 2031 | 772.8 | 106.8 (49.3 - 211.4) | 13.8 (6.4 - 27.4) | 363.6 | 63.7 (29.4 - 121.3) | 17.5 (8.1 - 33.4) | 409.2 | 37.0 (15.0 - 83.1) | 9.0 (3.7 - 20.3) |
| 2032 | 769.5 | 106.9 (47.7 - 216.7) | 13.9 (6.2 - 28.2) | 362.0 | 63.8 (28.5 - 124.2) | 17.6 (7.9 - 34.3) | 407.4 | 36.8 (14.3 - 85.0) | 9.0 (3.5 - 20.9) |
| 2033 | 766.0 | 106.3 (45.5 - 219.9) | 13.9 (5.9 - 28.7) | 360.4 | 63.5 (27.4 - 125.8) | 17.6 (7.6 - 34.9) | 405.6 | 36.5 (13.6 - 86.6) | 9.0 (3.4 - 21.3) |
| 2034 | 762.7 | 107.7 (44.8 - 227.0) | 14.1 (5.9 - 29.8) | 358.8 | 63.9 (26.8 - 128.8) | 17.8 (7.5 - 35.9) | 403.9 | 36.3 (13.2 - 88.2) | 9.0 (3.3 - 21.8) |
| 2035 | 759.7 | 107.3 (43.3 - 231.9) | 14.1 (5.7 - 30.5) | 357.5 | 64.1 (26.1 - 131.8) | 17.9 (7.3 - 36.9) | 402.2 | 35.1 (12.3 - 88.1) | 8.7 (3.1 - 21.9) |
| 2036 | 756.9 | 107.8 (42.2 - 237.9) | 14.2 (5.6 - 31.4) | 356.2 | 65.1 (25.7 - 135.9) | 18.3 (7.2 - 38.1) | 400.7 | 34.1 (11.5 - 88.0) | 8.5 (2.9 - 22.0) |
| 2037 | 753.6 | 107.6 (41.1 - 241.6) | 14.3 (5.5 - 32.1) | 354.7 | 65.1 (25.0 - 138.6) | 18.4 (7.1 - 39.1) | 398.9 | 33.1 (10.8 - 88.3) | 8.3 (2.7 - 22.1) |
| 2038 | 750.2 | 107.5 (39.7 - 245.1) | 14.3 (5.3 - 32.7) | 353.1 | 64.8 (24.2 - 141.0) | 18.3 (6.8 - 39.9) | 397.1 | 32.4 (10.3 - 88.9) | 8.2 (2.6 - 22.4) |
| 2039 | 746.6 | 106.2 (38.0 - 247.5) | 14.2 (5.1 - 33.1) | 351.5 | 64.2 (23.3 - 142.0) | 18.3 (6.6 - 40.4) | 395.1 | 31.7 (9.8 - 89.5) | 8.0 (2.5 - 22.6) |
| 2040 | 743.1 | 103.9 (36.0 - 249.2) | 14.0 (4.8 - 33.5) | 349.9 | 63.4 (22.3 - 142.9) | 18.1 (6.4 - 40.8) | 393.1 | 30.3 (9.1 - 89.1) | 7.7 (2.3 - 22.7) |
| BAPC, Bayesian age-period-cohort; CI, credible intervals.  BAPC model: The BAPC modeling with the R *bamp* package, where the future prevalence of diabetes is projected considering the age, period, and cohort effects using the random walks of first and second order (RW1 or RW2).  Age-specific observed diabetes prevalence among NHNS participants from 2003 to 2019 and national-level age-specific population projections from 2020 to 2040 in Japan were modeled in the BAPC to generate national-level age-specific diabetes projections. Age-specific projected prevalence data from the BAPC were then applied to national-level population projections data to estimate the age-aggregated prevalence at the national level.  The definition of diabetes corresponded to the official NHNS’s 'strongly suspected diabetes' category (ascertained by HbA1c% ≥6.5, or medication use), rather than strictly diagnosed diabetes. | | | | | | | | | |

**Supplementary Table 5**

| **Table S5: External validation RMSE values for predictive performance of BAPC and conventional models comparing the predicted prevalence (%) of diabetes mellitus with the official NHNS-reported prevalence across 2022-2024, Japan** | | | |
| --- | --- | --- | --- |
| **Population** | **RMSE** | | |
|  | **BAPC model** | **Conventional model 1** | **Conventional model 2** |
| **Total** | 0.26 | 0.34 | 0.29 |
| **Men** | 0.70 | 0.90 | 0.74 |
| **Women** | 0.29 | 0.56 | 0.78 |
| RMSE, root mean square error; BAPC, Bayesian age-period-cohort; NHNS, National Health and Nutrition Survey; and BAMP, Bayesian age-period-cohort modeling and prediction.  BAPC model: The BAPC modeling with the R *bamp* package, where the future prevalence of diabetes is projected considering the age, period, and cohort effects using the random walks of first and second order (RW1 or RW2).  Conventional model 1: The conventional prediction model with constant prevalence of diabetes, where the future prevalence is projected assuming a constant prevalence as observed in the last year of observed data (2019 in this study).  Conventional model 2: The conventional prediction model with monotonic changes in diabetes prevalence, where the future prevalence is projected by using the prevalence that monotonically changed according to the mean percentage changes observed from 2003 to 2019.  RMSE shows the annual average error in the prevalence (%) of diabetes mellitus predicted by the BAPC and conventional models relative to the official NHNS-reported prevalence across 2022-2024 in Japan.  The definition of diabetes corresponded to the official NHNS’s 'strongly suspected diabetes' category (ascertained by HbA1c% ≥6.5, or medication use), rather than strictly diagnosed diabetes. | | | |

**Supplementary Table 6**

| **Table S6. Trends in age-standardized projected prevalence (%) of diabetes mellitus at the national level derived from the BAPC model, Japan, from 2020 to 2040** | | | |
| --- | --- | --- | --- |
| **Year** | **Total** | **Men** | **Women** |
|  | **Median (95% CI)** | | |
| 2020 | 12.6 (9.6 - 16.4) | 17.0 (12.7 - 22.3) | 9.1 (6.4 - 12.7) |
| 2021 | 12.7 (8.9 - 17.6) | 17.1 (12.0 - 23.6) | 9.0 (5.9 - 13.5) |
| 2022 | 12.6 (8.4 - 18.6) | 17.0 (11.3 - 24.9) | 8.8 (5.3 - 14.0) |
| 2023 | 12.8 (8.1 - 19.7) | 17.0 (10.7 - 25.9) | 8.8 (5.0 - 14.8) |
| 2024 | 12.8 (7.7 - 20.7) | 17.2 (10.3 - 27.1) | 8.7 (4.7 - 15.4) |
| 2025 | 12.8 (7.4 - 21.5) | 17.2 (9.9 - 27.9) | 8.5 (4.4 - 15.7) |
| 2026 | 12.8 (7.1 - 22.3) | 17.2 (9.5 - 28.9) | 8.4 (4.1 - 16.2) |
| 2027 | 12.9 (6.9 - 23.2) | 17.4 (9.3 - 29.9) | 8.3 (3.8 - 16.6) |
| 2028 | 12.9 (6.5 - 23.8) | 17.4 (8.9 - 30.7) | 8.1 (3.6 - 16.9) |
| 2029 | 12.8 (6.2 - 24.3) | 17.3 (8.5 - 31.4) | 7.9 (3.4 - 17.2) |
| 2030 | 12.9 (6.0 - 25.1) | 17.4 (8.3 - 32.2) | 7.8 (3.2 - 17.5) |
| 2031 | 12.8 (5.9 - 25.7) | 17.3 (8.0 - 33.0) | 7.6 (3.0 - 17.6) |
| 2032 | 12.8 (5.6 - 26.3) | 17.3 (7.7 - 33.8) | 7.5 (2.9 - 18.0) |
| 2033 | 12.7 (5.4 - 26.7) | 17.3 (7.4 - 34.3) | 7.4 (2.7 - 18.3) |
| 2034 | 12.8 (5.3 - 27.6) | 17.4 (7.3 - 35.1) | 7.4 (2.6 - 18.6) |
| 2035 | 12.8 (5.1 - 28.2) | 17.4 (7.1 - 35.9) | 7.2 (2.4 - 18.6) |
| 2036 | 12.9 (5.0 - 29.0) | 17.6 (6.9 - 37.1) | 7.0 (2.3 - 18.7) |
| 2037 | 12.8 (4.8 - 29.5) | 17.6 (6.8 - 37.9) | 6.8 (2.2 - 18.9) |
| 2038 | 12.8 (4.7 - 29.9) | 17.5 (6.5 - 38.6) | 6.6 (2.1 - 18.9) |
| 2039 | 12.7 (4.5 - 30.2) | 17.4 (6.2 - 39.0) | 6.5 (1.9 - 19.1) |
| 2040 | 12.4 (4.2 - 30.6) | 17.2 (6.0 - 39.4) | 6.2 (1.8 - 19.1) |
| BAPC, Bayesian age-period-cohort; CI, credible intervals.  BAPC model: The BAPC modeling with the R *bamp* package, where the future prevalence of diabetes is projected considering the age, period, and cohort effects using the random walks of first and second order (RW1 or RW2).  Age-specific observed diabetes prevalence among NHNS participants from 2003 to 2019 and national-level age-specific population projections from 2020 to 2040 in Japan were modeled in the BAPC to generate national-level age-specific diabetes projections.  Age-standardized diabetes prevalence was calculated by the direct method using the 2015 Japan Standard Population. The national-level age-specific prevalence was weighted by the corresponding Standard Population weights. The weighted values were then summed and divided by the total Standard Population to obtain age-standardized prevalence.  The definition of diabetes corresponded to the official NHNS’s 'strongly suspected diabetes' category (ascertained by HbA1c% ≥6.5, or medication use), rather than strictly diagnosed diabetes. | | | |

**Supplementary Table 7**

| **Table S7. Calculated RMSE and trends in projected number of cases (×100,000) and prevalence (%) of diabetes mellitus at the national level derived from the BAPC model in sensitivity analysis using the age-effect smoothness prior of log(2.0) and the period and cohort priors of log(1.5), Japan, from 2020 to 2040** | | | | | | | | | |
| --- | --- | --- | --- | --- | --- | --- | --- | --- | --- |
| **Year** | **Total**  **(RMSE =** 27.6**)** | | | **Men**  **(RMSE =** 39.7**)** | | | **Women**  **(RMSE =** 12.3**)** | | |
|  | **Population**  **(×100,000)** | **Case**  **(×100,000)**  **Median (95% CI)** | **Prevalence**  **Median (95% CI)** | **Population**  **(×100,000)** | **Case**  **(×100,000)**  **Median (95% CI)** | **Prevalence**  **Median (95% CI)** | **Population**  **(×100,000)** | **Case**  **(×100,000)**  **Median (95% CI)** | **Prevalence**  **Median (95% CI)** |
| 2020 | 785.0 | 99.0 (75.6 - 128.8) | 12.6 (9.6 - 16.4) | 369.7 | 60.5 (45.3 - 79.8) | 16.4 (12.2 - 21.6) | 415.3 | 40.1 (28.5 - 56.3) | 9.7 (6.9 - 13.5) |
| 2021 | 785.9 | 99.7 (70.9 - 138.5) | 12.7 (9.0 - 17.6) | 370.2 | 60.7 (42.6 - 84.5) | 16.4 (11.5 - 22.8) | 415.7 | 40.2 (26.4 - 60.4) | 9.7 (6.4 - 14.5) |
| 2022 | 786.4 | 100.1 (66.6 - 147.5) | 12.7 (8.5 - 18.8) | 370.4 | 60.8 (40.1 - 89.5) | 16.4 (10.8 - 24.2) | 416.1 | 39.6 (24.1 - 63.8) | 9.5 (5.8 - 15.3) |
| 2023 | 786.7 | 102.1 (64.4 - 157.2) | 13.0 (8.2 - 20.0) | 370.5 | 61.3 (38.3 - 93.9) | 16.5 (10.3 - 25.3) | 416.3 | 40.2 (23.2 - 67.7) | 9.7 (5.6 - 16.3) |
| 2024 | 786.8 | 103.1 (62.0 - 165.5) | 13.1 (7.9 - 21.0) | 370.5 | 62.0 (37.2 - 98.4) | 16.7 (10.0 - 26.6) | 416.3 | 40.0 (21.7 - 70.3) | 9.6 (5.2 - 16.9) |
| 2025 | 786.3 | 103.3 (59.8 - 171.0) | 13.1 (7.6 - 21.7) | 370.2 | 62.2 (35.9 - 101.6) | 16.8 (9.7 - 27.4) | 416.1 | 39.9 (20.7 - 72.7) | 9.6 (5.0 - 17.5) |
| 2026 | 785.0 | 104.3 (57.8 - 178.9) | 13.3 (7.4 - 22.8) | 369.6 | 62.7 (34.7 - 105.4) | 17.0 (9.4 - 28.5) | 415.4 | 40.0 (19.8 - 76.1) | 9.6 (4.8 - 18.3) |
| 2027 | 783.4 | 105.1 (56.0 - 185.7) | 13.4 (7.1 - 23.7) | 368.8 | 63.5 (33.8 - 109.6) | 17.2 (9.2 - 29.7) | 414.6 | 39.6 (18.7 - 77.9) | 9.5 (4.5 - 18.8) |
| 2028 | 781.3 | 105.4 (53.9 - 192.9) | 13.5 (6.9 - 24.7) | 367.7 | 63.5 (32.6 - 113.1) | 17.3 (8.9 - 30.8) | 413.5 | 39.5 (17.9 - 80.8) | 9.5 (4.3 - 19.5) |
| 2029 | 778.9 | 104.2 (50.9 - 198.1) | 13.4 (6.5 - 25.4) | 366.6 | 63.1 (31.1 - 115.6) | 17.2 (8.5 - 31.5) | 412.3 | 38.4 (16.5 - 82.1) | 9.3 (4.0 - 19.9) |
| 2030 | 776.0 | 105.4 (49.9 - 205.9) | 13.6 (6.4 - 26.5) | 365.2 | 63.5 (30.4 - 118.3) | 17.4 (8.3 - 32.4) | 410.8 | 38.6 (16.1 - 84.0) | 9.4 (3.9 - 20.5) |
| 2031 | 772.8 | 105.6 (48.7 - 211.2) | 13.7 (6.3 - 27.3) | 363.6 | 63.2 (29.3 - 121.1) | 17.4 (8.1 - 33.3) | 409.2 | 38.2 (15.3 - 86.4) | 9.3 (3.7 - 21.1) |
| 2032 | 769.5 | 106.0 (47.0 - 216.1) | 13.8 (6.1 - 28.1) | 362.0 | 63.1 (28.3 - 123.3) | 17.4 (7.8 - 34.1) | 407.4 | 38.1 (14.7 - 88.2) | 9.3 (3.6 - 21.6) |
| 2033 | 766.0 | 105.4 (45.3 - 220.0) | 13.8 (5.9 - 28.7) | 360.4 | 62.9 (27.4 - 125.1) | 17.5 (7.6 - 34.7) | 405.6 | 37.9 (14.0 - 90.2) | 9.3 (3.4 - 22.2) |
| 2034 | 762.7 | 106.5 (44.6 - 224.5) | 14.0 (5.8 - 29.4) | 358.8 | 63.3 (26.9 - 128.5) | 17.6 (7.5 - 35.8) | 403.9 | 38.0 (13.6 - 92.0) | 9.4 (3.4 - 22.8) |
| 2035 | 759.7 | 106.0 (43.0 - 228.2) | 14.0 (5.7 - 30.0) | 357.5 | 63.4 (26.1 - 132.2) | 17.7 (7.3 - 37.0) | 402.2 | 36.8 (12.6 - 92.5) | 9.1 (3.1 - 23.0) |
| 2036 | 756.9 | 106.8 (41.3 - 233.0) | 14.1 (5.5 - 30.8) | 356.2 | 64.1 (25.6 - 135.5) | 18.0 (7.2 - 38.1) | 400.7 | 35.8 (11.8 - 92.9) | 8.9 (2.9 - 23.2) |
| 2037 | 753.6 | 106.4 (39.4 - 238.0) | 14.1 (5.2 - 31.6) | 354.7 | 64.3 (25.1 - 138.1) | 18.1 (7.1 - 38.9) | 398.9 | 34.9 (10.9 - 92.2) | 8.8 (2.7 - 23.1) |
| 2038 | 750.2 | 105.8 (38.5 - 243.1) | 14.1 (5.1 - 32.4) | 353.1 | 63.9 (24.1 - 140.9) | 18.1 (6.8 - 39.9) | 397.1 | 34.2 (10.7 - 93.3) | 8.6 (2.7 - 23.5) |
| 2039 | 746.6 | 104.2 (37.0 - 245.0) | 14.0 (5.0 - 32.8) | 351.5 | 63.3 (23.2 - 143.2) | 18.0 (6.6 - 40.7) | 395.1 | 33.4 (10.0 - 93.2) | 8.4 (2.5 - 23.6) |
| 2040 | 743.1 | 102.3 (35.0 - 247.2) | 13.8 (4.7 - 33.3) | 349.9 | 62.5 (22.2 - 143.8) | 17.9 (6.3 - 41.1) | 393.1 | 32.0 (9.2 - 93.8) | 8.2 (2.3 - 23.9) |
| RMSE, root mean square error; and BAPC, Bayesian age-period-cohort; CI, credible intervals.  RMSE shows the annual average error in the total count of cases for observed data across 2015-2019.  BAPC model: The BAPC modeling with the R *bamp* package, where the future prevalence of diabetes is projected considering the age, period, and cohort effects using the random walks of first and second order (RW1 or RW2).  Age-specific observed diabetes prevalence among NHNS participants from 2003 to 2019 and national-level age-specific population projections from 2020 to 2040 in Japan were modeled in the BAPC to generate national-level age-specific and age-aggregated overall diabetes projections. Age-specific projected prevalence data from the BAPC were then applied to national-level population projections data to estimate the age-aggregated prevalence at the national level.  The definition of diabetes corresponded to the official NHNS’s 'strongly suspected diabetes' category (ascertained by HbA1c% ≥6.5, or medication use), rather than strictly diagnosed diabetes. | | | | | | | | | |

**Supplementary Table 8**

| **Table S8. Calculated validation RMSE and trends in projected prevalence (%) of diabetes mellitus in selected projection years at the national level derived from the BAPC model in sensitivity analysis according to some selected MCMC iteration and tuning setting combinations following population down-sampling, Japan, from 2020 to 2040** | | | | | |
| --- | --- | --- | --- | --- | --- |
| **Sex** | **Iteration and tuning setting combination** | **Validation RMSE**  **(Prevalence % points)** | **Prevalence (median)** | | |
|  |  |  | **2020** | **2030** | **2040** |
| Total | Half iteration and half tuning | 0.216 | 12.6 | 13.6 | 13.7 |
| Men | Half iteration and half tuning | 0.735 | 16.4 | 17.4 | 17.9 |
| Women | Full iteration and full tuning | 0.294 | 9.7 | 9.0 | 7.6 |
| RMSE, root mean square error; and BAPC, Bayesian age-period-cohort.  Down-sampling methods involve one-quarter of the population and cases, deliberately dividing the age-specific population and case numbers by 4. Because NHNS’s expanded surveys in 2012 and 2016 were conducted with approximately 4 times the population size as in other usual survey cycles.  Validation RMSE (expressed as a prevalence percentage) was calculated as the annual average difference between the BAPC-projected DM prevalence using down-samples and the official NHNS-reported DM prevalence across 2022-2024, separately for the total and for each sex group, and for each iteration-tuning combination.  BAPC model: The BAPC modeling with the R *bamp* package, where the future prevalence of diabetes is projected considering the age, period, and cohort effects using the random walks of first and second order (RW1 or RW2).  Age-specific observed diabetes prevalence among NHNS participants from 2003 to 2019 and national-level age-specific population projections from 2020 to 2040 in Japan were modeled in the BAPC to generate national-level age-specific and age-aggregated overall diabetes projections. Age-specific projected prevalence data from the BAPC were then applied to national-level population projections data to estimate the age-aggregated prevalence at the national level.  The definition of diabetes corresponded to the official NHNS’s 'strongly suspected diabetes' category (ascertained by HbA1c% ≥6.5, or medication use), rather than strictly diagnosed diabetes. | | | | | |

**Supplementary Figures**

**Supplementary Figure 1**

Figure S1 illustrates the median (solid lines) and 95% credible intervals (shading) for the age effects coefficients obtained from the Bayesian age-period-cohort model, which estimates future trends in diabetes prevalence in Japan from 2020 to 2040.

**Supplementary Figure 2**

Figure S2 illustrates the median (solid lines) and 95% credible intervals (shading) for the period effects coefficients derived from the Bayesian age-period-cohort model, which estimates future trends in diabetes prevalence in Japan from 2020 to 2040.

**Supplementary Figure 3**

Figure S3 illustrates the median (solid lines) and 95% credible intervals (shading) for the cohort effects coefficients derived from the Bayesian age-period-cohort model, which estimates future trends in diabetes prevalence in Japan from 2020 to 2040. Birth cohort (index) represents the sequential birth cohort numbers derived from the BAPC model. Higher index values (on the right) correspond to younger birth cohorts, while lower values (on the left) represent older cohorts.

**Supplementary Figure 4**

Figure S4 illustrates the decomposition of observed (fitted) and projected changes in the crude prevalence of diabetes mellitus in Japan from 2003 to 2040 into contributions from population aging (changes in the demographic age structure) and age-specific prevalence changes, along with their interaction. The Bayesian age-period-cohort posterior fitted estimates were anchored to observed National Health and Nutrition Survey data (2003-2019) and posterior predictive estimates projected from the model (2020-2040).

Annual DM prevalence was calculated as the population-weighted average of age-specific prevalence estimates, based on national population projections for 9 age groups.

**References:**

1.	Schmid, V.J. and L. Held, *Bayesian Age-Period-Cohort Modeling and Prediction - BAMP.* Journal of Statistical Software, 2007. **21**(8): p. 1 - 15.

2.	Guzman Castillo, M., et al., *Future declines of coronary heart disease mortality in England and Wales could counter the burden of population ageing.* PLoS One, 2014. **9**(6): p. e99482.

3.	Pearson-Stuttard, J., et al., *Modeling Future Cardiovascular Disease Mortality in the United States: National Trends and Racial and Ethnic Disparities.* Circulation, 2016. **133**(10): p. 967-78.

4.	Kiyoshige, E., et al., *Projections of future coronary heart disease and stroke mortality in Japan until 2040: a Bayesian age-period-cohort analysis.* Lancet Reg Health West Pac, 2023. **31**: p. 100637.

5.	Knorr-Held, L. and E. Rainer, *Projections of lung cancer mortality in West Germany: a case study in Bayesian prediction.* Biostatistics, 2001. **2**(1): p. 109-29.

6.	Goldstein, H., *Age, Period And Cohort Effects — A Confounded Confusion.* Journal of Applied Statistics, 1979. **6**(1): p. 19-24.
